# Consumer-expressed needs concerning age-friendly products in China: a computational grounded theory analysis of online reviews

**DOI:** 10.3389/fpubh.2026.1869463

**Published:** 2026-09-09

**Authors:** Zhengze Li, Zhenkang Fu, Qinghua Zhu, Kejia He

**Affiliations:** School of Information Management, Nanjing University, Nanjing, China

**Keywords:** age-friendly, BERTopic, computational grounded theory, online review, user needs model

## Abstract

**Purpose:**

As population aging in China continues to intensify, age-friendly products play an increasingly important role in supporting older adults’ quality of life and advancing the smart senior care industry. However, existing studies predominantly rely on traditional methods, such as questionnaires and interviews, to identify user needs, with limited systematic investigation of needs expressed in real-world product contexts. To address this gap, this study analyzes consumer expressions in online reviews from Chinese e-commerce platforms and adopts computational grounded theory to identify and conceptualize user needs concerning age-friendly products.

**Methods:**

A total of 17,133 reviews concerning 66 age-friendly products were collected from Taobao and Tmall. The corpus may include reviews written by older product users, family purchasers, caregivers, institutional staff, and other consumers. After data preprocessing, a computational grounded theory approach was applied through a three-stage analytical procedure comprising pattern detection, pattern refinement, and pattern confirmation.

**Results:**

The analysis identified 8 main categories and 28 basic categories of user needs derived from consumer reviews concerning age-friendly products. These categories were organized into a four-layer conceptual framework comprising the foundational assurance layer, core utility layer, adaptation support layer, and value-oriented vision layer.

**Conclusion:**

By analyzing naturally occurring online reviews, this study broadens the data sources and contextual scope of user needs research concerning age-friendly products. The four-layer framework distinguishes the complementary roles of assurance, practical utility, adaptation support, and value evaluation across product acquisition, everyday use, caregiving, and service contexts. The application of computational grounded theory to user-generated content not only provides a systematic approach for uncovering latent user needs, but also offers actionable insights for the design and optimization of age-friendly products, as well as for the advancement of the smart senior care industry.

## 1 Introduction

By the end of 2025, China’s population aged 60 and above is projected to reach 323.38 million, accounting for 23.0% of the total population, while those aged 65 and above will reach 223.65 million, representing 15.9%. China is expected to enter a phase of severe population aging by 2035, which will substantially increase the demand for age-friendly products. In response to this demographic shift, the Chinese government has placed significant emphasis on the development of age-friendly products. For instance, in July 2025, the Ministry of Industry and Information Technology (MIIT) issued the “General Technical Requirements for Smart Senior Care Home Products” to standardize technical specifications and promote the integration of age-friendly and intelligent features into home products. In addition, the MIIT released the “2025 Catalog of Recommended Senior Care Products” in the same year to accelerate the adoption of high-quality age-friendly products in the daily lives of older adults, thereby supporting the development of an age-friendly society.

In this context, the effective use of age-friendly products has become increasingly important because these products are intended to support older adults’ daily activities, health-related needs, and independent living (1, 2). With the growing prominence of age-friendly design and smart senior care, research on age-friendly products has gained momentum. However, existing studies tend to focus either on micro-level functional design or on macro-level theoretical frameworks (3), with less attention to the broader set of needs arising across product acquisition, use, caregiving, and service contexts. A limited number of studies have explored user needs through questionnaires, literature reviews, or analyses of particular product categories (4–7). These approaches provide important insights but remain constrained by relatively limited data sources and research perspectives. Consequently, it remains challenging to develop an integrated conceptual framework of user needs concerning age-friendly products.

Analyzing consumer reviews of mainstream age-friendly products through computational grounded theory provides a valuable approach to addressing these limitations. With the rapid expansion of e-commerce platforms, consumers increasingly describe product functionality, quality, purchasing, caregiving, and usage experiences in online reviews. Compared with literature reviews and questionnaire surveys, online reviews offer large data volumes, rapid updates, and contextual embeddedness in product-related experiences, making them a useful complementary source for identifying user needs (8, 9). Computational grounded theory, as a methodological framework that integrates computational data processing capabilities with researchers’ domain expertise, mitigates certain limitations of traditional grounded theory and extends its applicability to large-scale textual data analysis (10). In this study, consumer-expressed characterizes the source of the review data, whereas user needs describes the theoretical object identified and organized through conceptual analysis. Accordingly, the study derives user needs concerning age-friendly products from a mixed corpus that may include older product users, family purchasers, caregivers, institutional staff, and other consumers. Specifically, this study addresses the following research questions: What user needs concerning age-friendly products can be derived from consumer expressions in online reviews? How can these needs be organized within a structured conceptual framework?

This study draws on consumer review data collected from Taobao and Tmall—China’s largest e-commerce platforms—and employs a computational grounded theory approach. The dataset consists of 17,133 reviews of 66 age-friendly products. First, the BERTopic model is applied to identify latent thematic structures within the review data, based on which representative review texts are selected. Grounded coding is then used to identify, conceptualize, and organize user needs derived from the review texts. Finally, a supervised machine learning model assesses the linguistic distinguishability and cross-product classification consistency of the resulting main categories. Through this process, the study develops a four-layer conceptual framework of user needs concerning age-friendly products. In doing so, it addresses the limitations of prior research in terms of sample size and systematic identification of user needs. Furthermore, this study provides theoretical and practical insights for advancing computational grounded theory in the field of age-friendly design and for optimizing the functional design of age-friendly products.

## 2 Related works

### 2.1 Age-friendly products and user needs of older adults

As global population aging continues to intensify, improving the quality of life of older adults through technological and product innovation has become a critical concern for both academia and industry. Technologies and products designed to meet the needs of older adults are commonly referred to as “gerontechnology,” which aims to support independent living, health management, and social participation through technological means (11, 12). Against the backdrop of rapid advancements in smart senior care and digital health, age-friendly products have evolved into diverse forms, including care assistance devices, health monitoring devices, and companion-oriented smart products (6, 13–15). Existing studies indicate that well-designed age-friendly technologies can enhance convenience and perceived safety in daily life, while also helping to alleviate social pressures associated with population aging (16).

In this study, age-friendly products refer to products that are specifically designed, adapted, or marketed to accommodate age-related changes and to support older adults’ daily living, mobility, health management, safety, care, or social participation. Smart senior care refers more specifically to digitally enabled products and services that use sensing, connectivity, data processing, or intelligent functions to support these activities and care processes. Assistive technologies primarily maintain or improve functioning and participation for people with functional limitations, whereas age-friendly products constitute a broader category that may include both assistive devices and mainstream products explicitly adapted for age-related or caregiving contexts. General consumer products were included only when their title, description, intended users, or stated usage scenario demonstrated an explicit age-friendly or eldercare context (1, 11, 12).

With regard to user needs, existing research consistently shows that older adults exhibit distinct usage characteristics compared with other age groups. On the one hand, age-related declines in sensory and motor functions lead older users to place greater emphasis on usability factors, such as interface readability, interaction simplicity, and reduced operational burden (17, 18). On the other hand, older users also demonstrate pronounced emotional and social needs when interacting with technological products, including preferences for companionship, social connection, and psychological support (19). In addition, technology acceptance among older adults is influenced not only by product functionality and usability but also by individual factors such as digital literacy, self-efficacy, and social support (20, 21). Therefore, the design of age-friendly products should comprehensively address the multidimensional needs of older users across physiological, psychological, and social domains.

In terms of research methods, studies on age-friendly products and older adults’ needs have predominantly relied on traditional approaches, such as questionnaires, interviews, and experimental studies. For instance, in-depth interviews and focus groups have been used to identify barriers and needs encountered by older adults when using digital devices (17), while participatory design methods involve older users in the product development process to enhance usability and acceptance (22). Some studies have also employed behavioral and neuroexperimental data to objectively evaluate the effectiveness of age-friendly designs (23). Furthermore, research in human–computer interaction has proposed design principles tailored to older users to improve accessibility and user experience (24, 25). However, despite these contributions, such studies are often constrained by small sample sizes and context-specific experimental settings, leading to potential sample bias and limited generalizability. As a result, they struggle to capture the diverse and dynamic expressions of user needs in real-world usage contexts. In contrast, an increasing number of studies in other fields have begun to leverage large-scale user-generated data to identify user needs at scale and in naturally occurring contexts (26, 27).

### 2.2 User needs research based on online reviews

With the rapid development of e-commerce platforms and social media, users increasingly express their evaluations of product features, quality, and user experience through online reviews, which constitute a typical form of user-generated content (UGC). Compared with traditional survey and interview data, online reviews are characterized by large data volumes, rapid updates, and contextual embeddedness in product-related experiences. These advantages address key limitations of traditional research methods and have made online reviews an increasingly important data source for studying user experience and identifying user needs (28, 29). Existing studies suggest that online reviews not only reflect consumers’ evaluations of product attributes but also reveal latent user needs in real-world usage contexts, thereby providing valuable insights for product design and marketing decisions (30). In recent years, advances in natural language processing and data mining have further enabled the systematic analysis of online review data, promoting the development of data-driven approaches to user needs research (31). Nevertheless, these advantages do not make online reviews inherently objective or representative, as voluntary posting and unequal access to e-commerce platforms may introduce selection and measurement biases.

In terms of research methods, existing studies primarily rely on text mining and natural language processing techniques to analyze review data. Among these, sentiment analysis is one of the earliest and most widely used approaches, focusing on evaluating user satisfaction with different product attributes by identifying emotional tendencies in review texts (32). For example, sentiment analysis can be used to identify positive and negative evaluations of product features and to determine key attributes of concern to users (33). In addition, topic modeling methods have been widely applied to online review analysis. Latent Dirichlet Allocation (LDA), for instance, can automatically extract latent topic structures from large-scale text data, thereby uncovering core user needs (34, 35). More recently, deep learning–based methods have emerged, particularly those built on pre-trained language models. Models such as BERT and its variants can capture semantic information more accurately, improving the precision of review analysis (36, 37). Building on these advances, newer approaches such as BERTopic integrate Transformer-based semantic embeddings with clustering algorithms to achieve higher-quality topic identification (38, 39).

In terms of application domains, online review mining has been widely applied in product design and consumer behavior research. Prior studies have leveraged review data to identify key product attributes and map them into design parameters, thereby supporting product optimization and innovation (40). Other research has constructed user needs models based on review data to characterize the evolution of user needs, providing a basis for iterative product development and decision-making (41). Furthermore, online review analysis has been applied in domains such as smart products, mobile applications, and digital services to evaluate user experience and identify usage issues (42). For example, app store reviews have been analyzed to assess the usability of AI applications and to identify experience deficiencies in real-world usage contexts (43). These findings demonstrate that online review data not only captures users’ immediate evaluations of products but also reveals latent user needs and potential directions for improvement.

Despite these advances, several limitations remain in existing research on online review mining. On the one hand, many studies focus on single analytical methods, such as sentiment analysis or topic modeling, and lack in-depth interpretation of the underlying structure of user needs embedded in review texts. On the other hand, online review analysis is often dominated by quantitative techniques, with limited attention to the relationships and structural organization of user needs. As a result, recent studies have begun to integrate text mining with qualitative approaches to develop structured user needs models, enabling a more systematic understanding of user needs (44). In line with this trend, combining machine learning methods with qualitative analysis provides a promising direction for the in-depth exploration of user needs.

### 2.3 Computational grounded theory

Grounded theory, originally proposed by Glaser and Strauss, is a qualitative research method that generates theory inductively from empirical data through a systematic coding process. This approach emphasizes constant comparison throughout open, axial, and selective coding to identify concepts, categories, and their relationships, ultimately leading to the development of an interpretive theoretical framework (45). Owing to its capacity to derive theory directly from data, grounded theory has been widely applied across social science disciplines, including sociology, management, and information systems (46). However, with the rapid expansion of internet technologies and digital platforms, the volume of available textual data has increased substantially. In this context, traditional grounded theory, which relies heavily on manual coding, faces significant challenges in handling large-scale datasets, including low efficiency, high labor costs, and potential subjectivity in coding processes (47).

To address these challenges, Nelson proposed the Computational Grounded Theory (CGT) framework, which integrates machine learning and text mining techniques into the grounded theory process. This approach improves the efficiency of large-scale text analysis while preserving the interpretive depth of qualitative research (48). Within the CGT framework, analysis typically involves an iterative combination of computational processing and human interpretation. Specifically, computational methods are first employed to identify latent patterns or thematic structures in large-scale text data. Based on these intermediate outputs—such as topic clusters or document classifications—researchers then conduct manual coding and interpretive analysis to refine, categorize, and theorize user-relevant concepts. Finally, machine learning and statistical techniques are used to validate the robustness and generalizability of the derived findings, ensuring both analytical rigor and interpretive validity (47). This methodological framework integrates the pattern detection capabilities of computational tools with researchers’ theoretical interpretive abilities, enabling researchers to conduct systematic theoretical exploration within large-scale textual data and to systematically construct the structural relationships among user needs through the grounded coding process.

In recent years, with advances in natural language processing and computational social science, CGT has been increasingly applied in areas such as social media analysis, online communities, and digital society research. For example, prior studies have combined text mining with qualitative coding to identify and interpret public discourse and social issues in social media data (49), while others have used computational techniques to support grounded theory analysis and improve the efficiency of qualitative research in large-scale data environments (50). In addition, some studies have applied CGT to examine the development of China’s old-age social welfare system (51). These studies demonstrate that CGT can enhance analytical efficiency while maintaining interpretive depth, highlighting its strong potential for application in social science research (48, 49). Furthermore, the development of big data and text mining technologies has provided new data sources and analytical approaches for social science research (52). However, despite its growing use in social media and online community research, the application of CGT in product review analysis and in studies of user needs for age-friendly products remains limited.

### 2.4 Research gaps

In summary, although existing research has generated substantial insights into age-friendly products, online review mining, and text analysis methods, several limitations remain. First, prior studies on age-friendly products have predominantly emphasized product design principles and technology acceptance, while systematic investigations of how different types of user needs can be structurally organized remain limited. Second, most studies continue to rely on questionnaires and interviews, which may capture fewer naturally occurring expressions embedded in product acquisition, caregiving, use, and service encounters. Third, although online review mining has been widely applied, many studies rely on a single analytical technique and lack integration between computational pattern detection and qualitative theoretical analysis.

To address these limitations, this study analyzes consumer reviews of age-friendly products using computational grounded theory. Topic modeling supports the identification and screening of relevant review texts, while grounded coding identifies and conceptualizes user needs and organizes them into a structured framework. In this study, user needs refer broadly to product-related requirements expressed across acquisition, operation, caregiving use, and service support. Thus, consumer-expressed characterizes the source of the review data, whereas user needs characterizes the theoretical object of the analysis. The framework encompasses functional and psychological demands as well as contextual conditions that enable or constrain effective product use; service provision, logistics processes, and product quality are included when they shape usability, accessibility, reliability, or value in age-friendly product contexts. This approach supports the development of a conceptual framework of user needs concerning age-friendly products and offers practical implications for product and service optimization.

## 3 Methodology

### 3.1 Research framework

**Figure 1:**
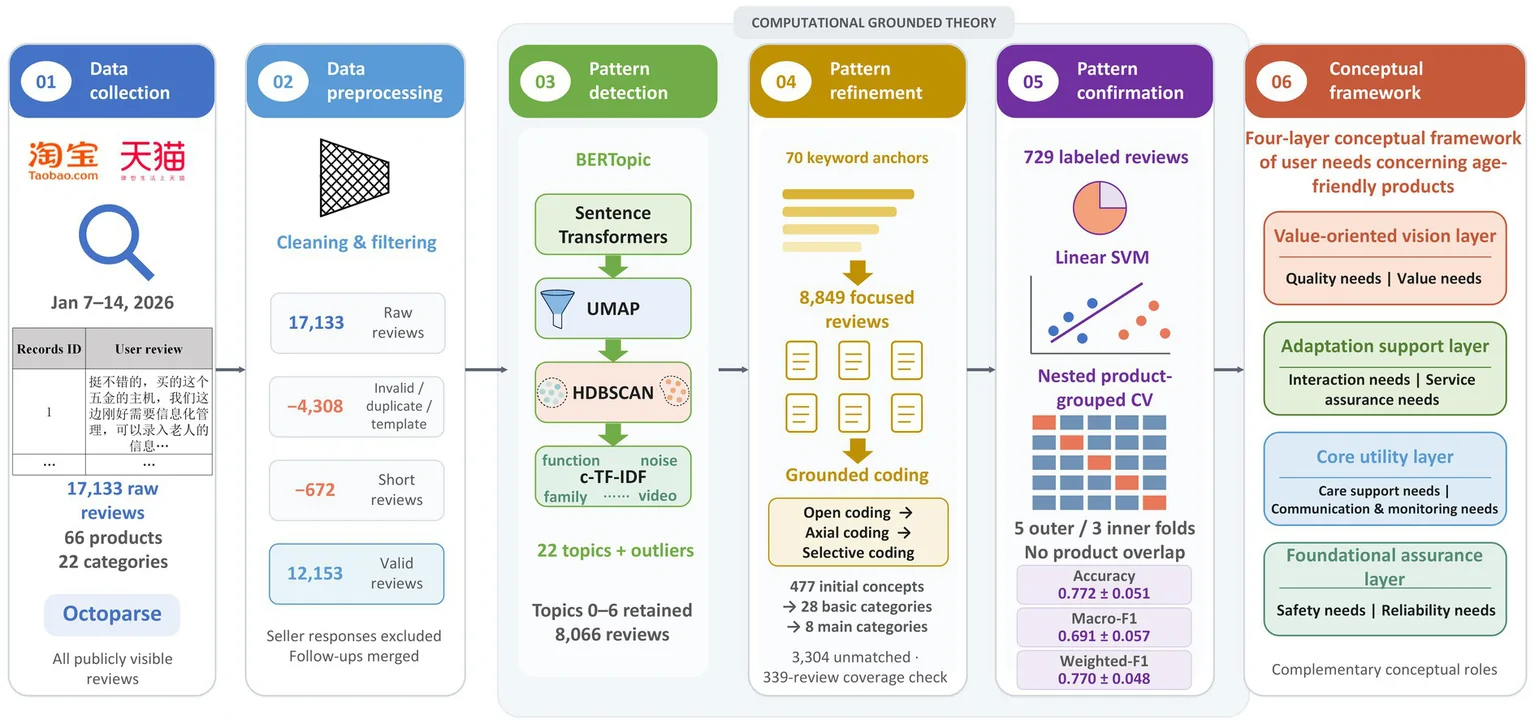
Research framework.

This study examines consumer reviews concerning age-friendly products in real-world product contexts to identify and organize user needs and to uncover potential shortcomings in current mainstream products. To achieve this objective, a research framework based on computational grounded theory is developed. The research process consists of six phases: data collection, data preprocessing, pattern detection, pattern refinement, pattern confirmation, and the development of a four-layer conceptual framework of user needs concerning age-friendly products. The overall research framework is illustrated in Figure 1.

### 3.2 Data collection

The study data were obtained from Taobao and Tmall, two major Chinese e-commerce platforms containing extensive feedback on age-friendly products. Data collection was conducted from January 7 to January 14, 2026, using Octoparse, a commercial web-scraping tool. The sampling frame was fixed on January 7, 2026, and covered all publicly visible reviews posted between July 17, 2025 and January 7, 2026 for the selected products. All accessible reviews were collected without imposing a maximum number of review pages or reviews per product. Reviews were not sampled or filtered according to platform relevance ranking or chronological display order; all reviews publicly visible by the cutoff date were collected irrespective of their display position. The retained fields included review text, review date, product information, product category, seller information, and follow-up-review status; account identifiers and product ratings were not retained. Collection was restricted to publicly accessible pages, involved no access to private or password-protected information, and followed applicable platform requirements and research-ethics principles. The unit of analysis was the individual online review. Reviewer age, identity, relationship to the product user, and level of direct product experience were not consistently available. The corpus may therefore include reviews written by older product users, family purchasers, informal or professional caregivers, institutional staff, and other consumers. Because these roles could not be reliably determined from the review text, reviews were not classified by reviewer type. Accordingly, the analysis derives user needs concerning age-friendly products from a mixed consumer review corpus; it does not assume that the corpus directly or representatively captures older adults’ first-person needs. To obtain broad coverage of products and reviews, we deployed a three-stage search strategy.

In the first stage, six primary Chinese search queries were derived from the literature and expert consultation: “适老化产品” (age-friendly product), “适老化设备” (age-friendly device), “智慧养老产品” (smart senior care product), “智慧养老设备” (smart senior care device), “智能养老产品” (intelligent older adults care product), and “智能养老设备” (intelligent older adults care device). This preliminary search identified 25 eligible products. All publicly visible reviews for these products were collected, yielding a foundational corpus of 5,211 reviews. These reviews were included in, rather than added separately to, the final corpus of 17,133 reviews.

In the second stage, BERT-based word embedding was used to expand the search vocabulary and reduce the semantic limitations of the initial queries (53). Using “age-friendly” “smart senior care” “intelligent older adults care” “product” and “device” as seed terms, a pre-trained BERT model generated word vectors for content words in the collected reviews. Cosine similarity was used to retrieve the five terms most semantically similar to each seed term. Three domain experts independently evaluated the candidate terms for semantic relevance and retrieval effectiveness, and disagreements were resolved through discussion. The retained first-group terms were “适老化,” “智慧养老,” “智能养老,” “智慧化养老,” “智能化养老,” “助老,” and “康养”; the second-group terms were “产品,” “设备,” “商品,” “物品,” and “设施.” Pairwise combination of the seven first-group terms and 5 s-group terms produced 35 Chinese search combinations (Keywords and grouping rules are listed in Supplementary Table 1).

In the third stage, the retrieved products were subjected to search filtering, category consolidation, and iterative sample expansion. Search filtering referred to the application of explicit inclusion and exclusion criteria. A product was included when its title, description, intended users, or stated usage scenario indicated that it was designed for older adults, individuals with age-related functional limitations, or caregiving and eldercare contexts. Irrelevant results, general consumer products without an explicit age-friendly context, duplicate product records, products without accessible textual reviews, and products whose primary purpose was unrelated to age-friendly use were excluded. Members of the research team independently assessed product eligibility and compared their decisions; disagreements were resolved through discussion until consensus was reached.

Category consolidation referred to grouping eligible products according to their primary functions and usage scenarios. Products with different commercial names but equivalent core functions were assigned to the same category, whereas products serving substantively different care purposes were retained as separate categories. Snowball sampling referred to an iterative expansion procedure in which related-product recommendations, functionally similar products, and additional product terms encountered during screening were used to conduct further searches. Newly identified eligible products were added to the sampling frame, and the process continued until repeated searches no longer yielded substantively new product categories. This procedure identified 66 types of age-friendly products across 22 functional categories and yielded 17,133 raw reviews. The category-level distribution is reported in Supplementary Table 2.

Review volumes across the 66 products ranged from 3 to 2,566, with a median of 47.5 and an interquartile range of 16–236.5. The most frequently reviewed product accounted for 14.98% of the corpus, indicating that no single product alone dominated the dataset.

### 3.3 Data preprocessing

The review data were preprocessed to improve the quality and integrity of subsequent text analysis. The data cleaning process involved four steps.

(1) Invalid review removal.

We removed Platform-generated default reviews (e.g., “This user rated the product as excellent, giving it 5 stars”), entries without textual feedback (e.g., “This user did not write a review”), and template-based content using rule-based matching. Exact duplicate reviews were identified through direct text matching, and only one instance of each duplicated text was retained. Examination of the duplicate records showed that identical texts primarily consisted of repeated platform-generated or promotional templates. Follow-up reviews were merged with their corresponding original reviews and treated as a single review record. Because account identifiers were not retained, repeated contributions from the same account could not be assessed. Reviews without explicit indicators of fabrication or incentivization were not subjectively classified as inauthentic. This stage removed 4,308 reviews, leaving 12,825 reviews.

(2) Short text filtering.

Reviews containing no more than five Chinese characters or consisting solely of punctuation were removed because such texts generally lacked sufficient information for topic extraction and grounded analysis. This stage removed 672 reviews, leaving 12,153 reviews. To examine whether this rule excluded concise but meaningful information, all 672 removed reviews were manually re-examined. Among them, 658 (97.92%) lacked identifiable product-, service-, safety-, or usage-related information. The remaining 14 reviews (2.08%; 0.08% of the original corpus) expressed concise evaluations related to basic categories already represented in the retained corpus, including material craftsmanship, customer service professionalism, safety protection, power performance, noise control, call responsiveness, psychological security, portable design, and ease of use. No additional need category was identified through this sensitivity check.

(3) Non-text feature cleaning.

Special symbols, non-semantic characters, and emojis were removed during text normalization, while the accompanying textual content was retained. Because the analysis focused on semantic need categories rather than sentiment intensity, emojis were not independently treated as analytical features. Obvious misspellings were corrected according to context, and dialectal or colloquial expressions were replaced with standard Chinese equivalents only when their meanings were unambiguous. Meaningful English product names, model numbers, technical abbreviations, and other semantically relevant expressions were retained. These normalization procedures altered the textual representation but did not remove additional review records.

(4) Word segmentation and stopword filtering.

Chinese word segmentation and part-of-speech tagging were performed using the Paddle mode of Jieba 0.42.1 with PaddlePaddle-tiny 1.6.1, which was used to improve segmentation and tagging of informal e-commerce review text.

A multi-level vocabulary-filtering procedure was applied. First, Baidu’s stop-word list was used as the initial list and supplemented with domain-specific terms (e.g., “item” and “seller”). Negation terms, such as “不” (not), “没有” (not have), “不能” (cannot), and “不会” (do not know how to), were explicitly retained to preserve semantic polarity. Second, part-of-speech tagging was used to exclude particles, interjections, pronouns, and other terms with limited semantic contribution. Third, following conventional stop-word and part-of-speech filtering, a corpus-level TF-IDF matrix was constructed using the segmented reviews. For each term, its TF-IDF weight was averaged across all documents, including documents in which the term was absent. The vocabulary was ranked according to the mean TF-IDF weight, and terms within the lowest 10% of this distribution were removed as an additional corpus-specific vocabulary-filtering step. The 10% threshold was adopted as a conservative empirical cutoff that retained 90% of the corpus-specific vocabulary.

Data preprocessing was conducted using Python 3.9, Jieba 0.42.1, PaddlePaddle-tiny 1.6.1, scikit-learn 1.6.1, NumPy 1.23.5, and pandas 1.5.2. The preprocessing script and core code are provided as Supplementary materials to support reproducibility.

At the review-record level, 4,308 invalid, duplicate, or template-based reviews and 672 short reviews were removed, for a total of 4,980 excluded reviews. The final corpus therefore comprised 12,153 reviews containing 669,264 Chinese characters. Subsequent normalization, segmentation, and vocabulary filtering modified the textual representation but did not remove additional review records.

## 4 Results

### 4.1 Pattern detection

BERTopic was applied to detect latent thematic patterns in the complete corpus of 12,153 reviews. BERTopic combines pretrained language models with clustering techniques and is suitable for short and linguistically diverse review texts (44). During text vectorization, ‘moka-ai/m3e-base’, a Chinese-language sentence-embedding model, was used to generate semantic representations of the reviews. Sentence embeddings were reduced using UMAP with ‘n_neighbors = 30’, ‘n_components = 10’, ‘metric = cosine’, and ‘random_state = 42’. HDBSCAN was configured with ‘min_cluster_size = 50’, ‘metric = euclidean’, ‘cluster_selection_epsilon = 0.0’, and ‘prediction_data = True’. Representative topic keywords were subsequently generated using c-TF-IDF. The analysis was conducted using BERTopic 0.17.3, SentenceTransformers 3.3.1, umap-learn 0.5.11, and HDBSCAN 0.8.41.

The model generated 22 non-outlier topics. Of the 12,153 input reviews, 9,506 (78.22%) were assigned to Topics 0–21, while 2,647 (21.78%) were classified as outliers (`Topic −1`). The number of reviews in each topic and the topic-selection decisions are reported in Supplementary Table 3. The seven largest topics (Topics 0–6), containing 8,066 reviews, were retained as broad thematic anchors. Topics 7–9 were not retained because Topic 7 substantially overlapped with the battery and power content of Topic 1, Topic 8 consisted primarily of generic usage evaluations, and Topic 9 lacked sufficient semantic coherence. The remaining smaller topics were not retained as independent thematic anchors because their keywords mainly represented specific product categories or brands, generic evaluations, or content already captured by Topics 0–6.

**Figure 2:**
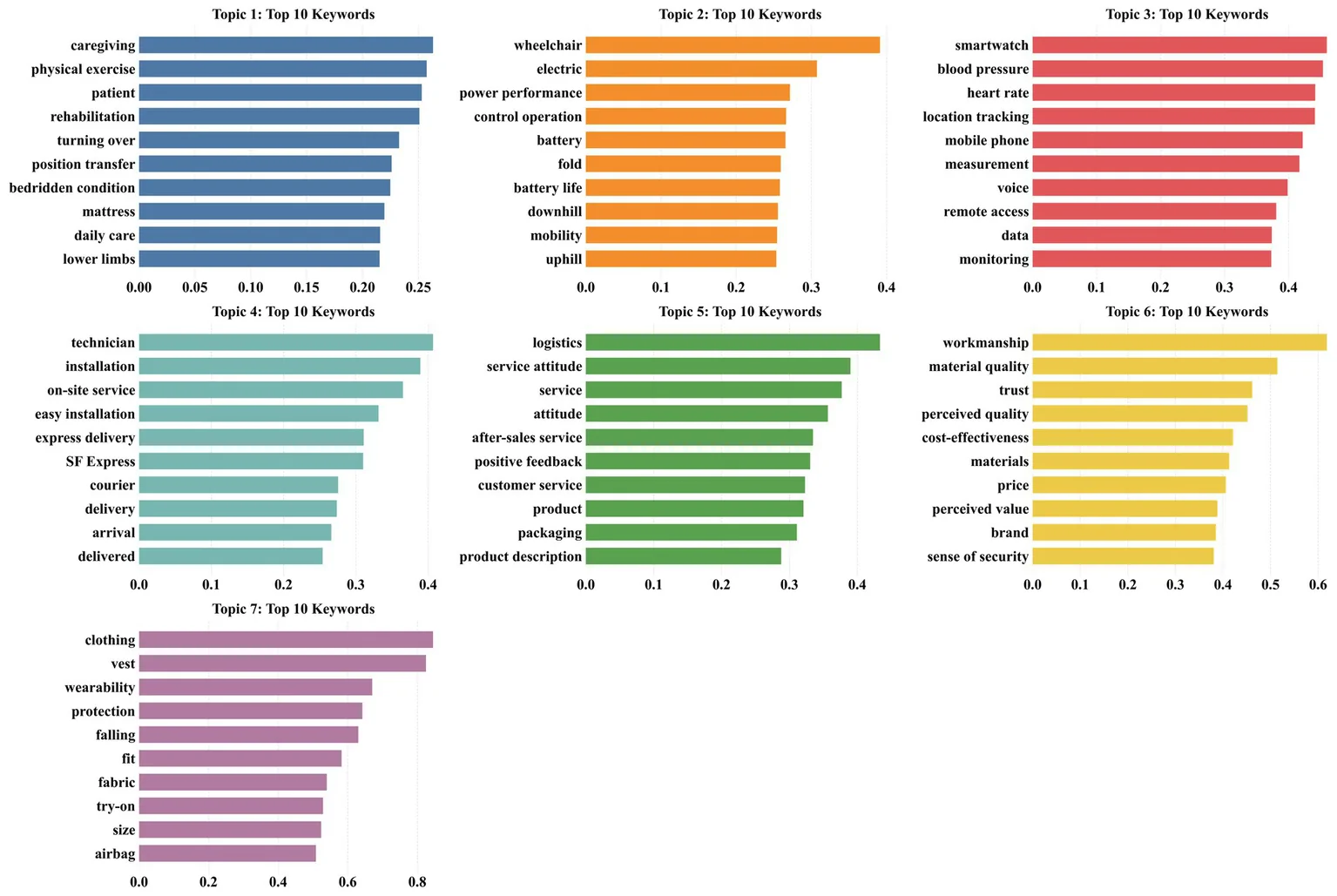
BERTopic keywords.

Several neighboring values of the UMAP and HDBSCAN parameters were iteratively compared. Candidate models were assessed according to topic distinctiveness, semantic interpretability of the keywords and associated reviews, fragmentation into overlapping clusters, and the proportion of outlier reviews. With the random seed fixed at 42, the broad thematic domains remained substantively consistent across the examined configurations, while the reported configuration produced the clearest separation among interpretable topics. Formal coherence scores were not used as the sole model-selection criterion; interpretability was assessed by three researchers who independently examined the keywords and associated review texts for all candidate topics. Their judgments were compared, and disagreements were resolved through discussion until consensus was reached. As a result, 7 meaningful topics were retained for subsequent analysis (Figure 2).

To reduce the risk of overlooking infrequent but important needs, three researchers additionally reviewed Topics 7–21 and the outlier cluster, with particular attention to safety failures, adverse events, and accessibility barriers. Although some smaller topics reflected product-specific contexts, such as stair mobility and toileting assistance, all substantive needs identified in these topics and outlier reviews could be accommodated within the 28 basic categories derived from the retained corpus. No additional need category emerged from this coverage check.

### 4.2 Pattern refinement

To construct a focused corpus for grounded coding, the top 10 keywords from each of the seven retained topics were used as 70 analytical anchors. Reviews were selected through exact substring matching rather than semantic-similarity retrieval. A review was included when its text contained at least one of the 70 keywords. Reviews containing multiple keywords were retained once as unique review records, while all matched keywords were recorded for contextual comparison. Applied to the complete corpus of 12,153 reviews, this procedure identified 8,849 unique reviews (72.81%) for grounded coding and left 3,304 reviews (27.19%) unmatched. The keyword-retrieved sample differed from the 8,066 reviews directly assigned to Topics 0–6 because matching was conducted across the complete corpus and could retrieve relevant reviews originally assigned to other topics.

The keyword-based procedure served as a computationally assisted corpus-screening step within the computational grounded theory workflow. It was used to construct a focused corpus for detailed and efficient grounded coding and was not treated as theoretical sampling in the classical grounded theory sense (48). Because exact keyword matching could overlook reviews expressing relevant needs through synonyms, implicit descriptions, uncommon language, or low-frequency expressions, unmatched reviews were not regarded as irrelevant or excluded from further consideration. Instead, they were retained as a complementary corpus for the subsequent coverage assessment.

As a *post-hoc* sensitivity analysis conducted after the grounded coding categories had been developed, 339 reviews were selected from the 3,304 unmatched reviews through product-stratified random sampling. This sample represented 10.26% of the unmatched corpus and covered all 62 products for which unmatched reviews were available. Comparison with the coding framework showed that the sampled reviews provided additional expressions and examples of product performance, rehabilitation support, usability, safety, and service experience, but these could be accommodated within the existing 28 basic categories. No conceptually distinct basic category or additional category relationship emerged from the coverage check, providing further evidence of conceptual saturation.

#### 4.2.1 Open coding

Open coding is a critical step in conceptualizing raw textual data, involving the systematic identification of concepts and extraction of coding elements through constant comparative analysis (46). In this study, coding was conducted in Chinese using Microsoft Excel. The two coders were a second-year master’s student and a third-year doctoral student in Information Resource Management, both of whom had substantial training and experience in grounded theory. They independently coded the complete focused corpus of 8,849 reviews. The coding framework was not established in advance. Instead, an initial batch of approximately 200 reviews was used to align the coders’ application of open coding and to develop an evolving codebook inductively from the data.

Reviews were processed in successive batches of approximately 200. Each review could receive multiple codes when it expressed more than one need. Constant comparison was conducted within and across batches by comparing review excerpts, initial codes, and emerging categories. Code definitions and representative examples were documented in the evolving Excel codebook. When new concepts or ambiguities emerged, code definitions and category boundaries were discussed and revised, and previously coded reviews were retrospectively checked against the updated definitions. This process generated 477 initial concepts, which were progressively refined and consolidated into 28 basic categories. Examples of the coding process are presented in Table 1.

**Table 1:** Examples of open coding.

Keywords	Representative texts	Initial concepts	Basic categories
mobile phone	The signal quality is excellent. There are three buildings, each with seven floors, and the main unit is placed in the first building, yet full signal coverage has been achieved. After 1 week of use, the signal has remained very stable, and the system also supports mobile phone notifications. Overall, it is a highly reliable system.	signal coverage	communication quality
easy installation	The wood grain is clear and the color is accurate, and the product features easy installation. We first saw the product at the seller’s exhibition and then customized a batch directly from the factory. The quality is excellent, and we received a wholesale price. The leadership team was very satisfied.	easy installation	ease of installation
position transfer	Since purchasing this device, position transfer for the older adult has become much easier. It is very comfortable to use and, when combined with the bed, is not only more comfortable than manual assistance but also significantly reduces physical effort. It allows convenient position transfer to the living room or bathroom and offers high value.	effort-saving position transfer	effort-saving effectiveness
price	Positive feedback! The product has been received, and the quality is excellent. More importantly, the price is affordable.	cost-effectiveness	price competitiveness
customer service	Placed under the bed sheet for the older adult, it has reduced concerns during the night compared to before. An alarm is triggered when the user sits up. The customer service attitude is excellent. I initially placed it incorrectly. Although it’s very simple, customer service patiently explained the logic to me. After adjustment, the system worked perfectly. After several days of use, there were no false alarms or missed alerts.	patient customer service	customer service professionalism
voice	The main unit has a loud voice output, but the volume is adjustable. At night, it can be lowered to facilitate rest, while during the day, it can be increased when more people are present without causing issues.	adjustable voice output	noise control
daily care	The device significantly improves efficiency in daily care, making it less time-consuming and physically demanding to care for the older adult.	reduced care burden	care convenience
battery life	After a period of use, the older user is very satisfied. The product has good quality, long battery life, and convenient features such as one-touch dialing and automatic call answering, which enhance usability.	long battery life	battery life
heart rate	After testing before reviewing. The ECG results match those from physical exams and the heart rate matches my self-measurements. It is impressive that such a compact device can maintain this level of performance.	accurate heart rate measurement	measurement accuracy
measurement	I bought this to take an ECG for my older adult when they were not feeling well, but the operation was so cumbersome and complicated that my parent could not figure it out at all. Even I, as a young person, found it complex—the results of the three-lead measurement could only be viewed on a computer, and alternative measurement methods (palm or ankle contact) are inaccurate. The product does not match its description and creates unnecessary frustration.	complex measurement process	measurement convenience
service	After my father was diagnosed with coronary heart disease, a cardiologist at a top-tier hospital recommended this device. It allows real-time monitoring at home. The operation is relatively simple and easy to understand. Reports can be exported and printed at any time—I can take them straight to the hospital and use them immediately; it’s so convenient. The one-year free service is also highly valuable.	exportable reports	reporting functionality
remote access	Concerned about my mother’s health, a doctor recommended purchasing a monitoring device. The customer service suggested this model, which is suitable. After connecting it to the network, real-time remote access is possible, and alerts are delivered promptly.	remote access capability	remote monitoring
sense of security	The flagship version has excellent workmanship and durable materials. When parents are happy and at ease, their children can also experience a stronger sense of security. Spending a few thousand yuan on this garment is like buying insurance for your parents—if they were to fall, the cost would far exceed a few thousand yuan.	sense of security	psychological security
fold	The product is of good quality, lightweight, and can be folded to fit easily into a car trunk. The older user enjoys using it for outings.	foldable storage	portable design
airbag	This product deserves recognition. I initially purchased it with some skepticism, but when the older adult fell a few days ago, the airbag deployed and prevented injury. It is a trustworthy product.	airbag deployment	safety protection
downhill	The intelligent brakes are very useful, so there is no need to worry about slipping when going uphill or downhill, which significantly enhances safety. Initially, I practiced using the first gear.	automatic braking function	safety braking

Conceptual saturation was assessed iteratively during the later stages of coding. Saturation was considered to have been reached when successive batches of approximately 200 reviews generated no new concepts, basic categories, or category relationships. The same two coders subsequently examined the product-stratified sample of 339 unmatched reviews described above; this additional coverage check yielded no conceptually distinct basic category or additional category relationship.

All computational analyses and grounded coding were conducted directly on the original Chinese review texts. English translation was undertaken only for reporting the findings and therefore did not affect topic detection or coding decisions. The review excerpts and category labels were initially translated by the two coders, with assistance from DeepL Pro. The fourth author independently checked the translations against the original Chinese texts for semantic equivalence, terminological consistency, and contextual accuracy. Any discrepancies were discussed with the coders and resolved by consensus. The English excerpts are faithful translations of the original Chinese reviews rather than interpretive paraphrases. The original Chinese texts and corresponding English translations of the review excerpts quoted in the manuscript are provided in Supplementary Table 4.

For quantitative contextualization, each review was assigned one primary main category according to its dominant semantic focus, while additional main-category and basic-category codes were retained when multiple needs were expressed. We calculated primary-category frequencies, total coding occurrences, the number of contributing products, coverage across the 22 age-friendly product categories, and pairwise main-category co-occurrence. Reviews spanning category boundaries were examined as discrepant cases through constant comparison. The primary category reflected the central evaluative emphasis of the review, while secondary codes preserved additional needs expressed in the same text.

#### 4.2.2 Axial coding

Axial coding integrates and clusters the initial concepts generated during open coding to reveal and refine more abstract category relationships (44). In this study, 28 basic categories were derived by abstracting the features and internal relationships among the initial concepts. These basic categories were further grouped into 8 main categories: quality needs, service assurance needs, reliability needs, care support needs, value needs, interaction needs, communication & monitoring needs, and safety needs. Consistent with the broad usage-context definition adopted in this study, the categories encompass functional and psychological demands as well as conditions that support effective product use. Together, they constitute the core components of the user needs framework concerning age-friendly products. Examples of the axial coding results are presented in Table 2.

**Table 2:** Results of axial coding.

Main categories	Basic categories	Definition of basic categories
Quality needs	noise control	The noise level during product operation
	packaging quality	The protective measures during transportation and the initial impression during unboxing
	portable design	The control of product size and the convenience of storage, adapting to both home and outdoor scenarios
	material craftsmanship	The product materials, workmanship details, and the tactile experience of user-contact surfaces
Service assurance needs	ease of installation	The simplicity of the installation process and the availability of professional support
	service reliability	The responsiveness of after-sales service and its ability to resolve problems
	customer service professionalism	The professional competence of customer service personnel
	logistics efficiency	The delivery speed and service details in the logistics process
Reliability needs	power performance	The output capability and stability of the core power system
	battery life	The duration of battery usage and the charging experience, determining continuity in daily use
	measurement accuracy	The professional reliability of health monitoring data
	data integration	The capability to exchange and manage data with external systems or devices
Care support needs	effort-saving effectiveness	The extent to which the product reduces the physical burden of caregivers
	care convenience	The practical design of daily care tasks for dependent older adults
	mattress comfort	The impact of contact surface materials on the comfort of long-term bedridden older adults
	functional integration	The degree and practicality of integrating multiple care functions within the product
Value needs	price competitiveness	The comparative advantage of price relative to similar products and its alignment with functionality and quality
	psychological security	The subjective emotional value provided by the product and its role in alleviating safety-related anxiety among older adults
Interaction needs	ease of use	The user-friendliness of interface and interaction design for older users
	measurement convenience	The simplicity of the health data collection process
	data visualization	The intuitiveness and readability of health data presentation
	reporting functionality	The capability of data export and report generation
Communication & monitoring needs	communication quality	The stability and accuracy of the communication module
	call responsiveness	The timeliness and accuracy of emergency call triggering and response
	remote monitoring	The capability for remote viewing and intervention
	multi-device synchronization	The ability to support multiple devices receiving information or being controlled simultaneously
Safety needs	safety protection	The effectiveness of proactive protection mechanisms in mitigating risks such as falls and collisions
	safety braking	The reliability and responsiveness of braking systems in mobility devices (wheelchairs, transfer devices)

#### 4.2.3 Selective coding

Selective coding is the process of analyzing the relationships among main categories to develop a theoretical storyline that explains the overall phenomenon (54). In this study, the core category is user needs concerning age-friendly products as derived from consumer reviews. Through further integration and comparative analysis of the 8 main categories, these categories were organized into a four-layer conceptual framework comprising the foundational assurance layer, core utility layer, adaptation support layer, and value-oriented vision layer. The four layers distinguish the complementary roles of user needs in product assurance, practical utility, usage adaptation, and value evaluation.

Specifically, safety and reliability needs constitute the foundational assurance layer, which concerns the safeguards supporting the use of age-friendly products. Care support needs and communication & monitoring needs form the core utility layer, reflecting the practical functions provided in care, monitoring, communication, and daily activities. Interaction needs and service assurance needs constitute the adaptation support layer, connecting product functions with operable and comprehensible usage experiences. Quality and value needs form the value-oriented vision layer, reflecting evaluations of product quality, psychological security, and long-term value. Together, these four complementary roles provide an integrated conceptual organization of user needs derived from consumer reviews concerning age-friendly products.

### 4.3 Pattern confirmation

Pattern confirmation was operationalized as an assessment of the linguistic distinguishability and cross-product classification consistency of the eight coded main categories. A total of 729 reviews were randomly sampled within the eight main categories to construct the labeled dataset. Although multiple codes were permitted during grounded coding, the supervised task was formulated as single-label multiclass classification at the main-category level. Each review was assigned the main category representing its dominant semantic focus. The final sample comprised 81 safety needs reviews, 105 quality needs reviews, 95 interaction needs reviews, 90 value needs reviews, 93 care support needs reviews, 77 communication & monitoring needs reviews, 99 service assurance needs reviews, and 89 reliability needs reviews.

Word-level TF-IDF features were extracted from the segmented and preprocessed review text, while character-level TF-IDF features were extracted from the original Chinese review text. The two representations were combined and classified using a linear support vector classifier implemented in scikit-learn 1.6.1. Hyperparameters were selected through a 40-configuration grid search covering word n-grams of (1, 2), character n-grams of (2, 4, 5), C values of 0.1, 0.5, 1.0, 2.0, and 5.0, and class weights of None and balanced. Macro-F1 was used as the selection criterion. The final configuration selected through product-grouped grid search used C = 0.5, balanced class weights, character 2-5-grams, and word unigrams.

Model evaluation used nested product-grouped cross-validation. The outer loop used five-fold stratified group cross-validation, and the inner loop used three-fold product-grouped grid search. Reviews from the same product were kept within a single fold. The 729 reviews represented 43 products, and no product appeared in both the training and validation sets within any outer fold. Across the five outer folds, mean accuracy was 0.772 (SD = 0.051), macro-F1 was 0.691 (SD = 0.057), micro-F1 was 0.772 (SD = 0.051), and weighted-F1 was 0.770 (SD = 0.048). Pooled outer-fold predictions yielded an accuracy of 0.742, macro-F1 of 0.749, and weighted-F1 of 0.743. Per-category results and the confusion matrix are reported in Supplementary Tables 5, 6.

The results indicate that the eight coded main categories exhibit identifiable linguistic patterns across products. Communication & monitoring needs and reliability needs showed the clearest separation, whereas safety needs, quality needs, and interaction needs displayed greater semantic overlap. Overall, the model demonstrated stable and reliable classification performance across age-friendly product reviews. These results support the reliability of the eight main categories and the robustness of the proposed four-layer conceptual framework.

### 4.4 Quantitative distribution and category coverage

Across the 8,849 coded reviews, service assurance needs constituted the largest primary category (*n* = 2,440, 27.6%), followed by care support needs (*n* = 1,876, 21.2%), interaction needs (*n* = 1,361, 15.4%), value needs (*n* = 1,213, 13.7%), quality needs (*n* = 1,056, 11.9%), safety needs (*n* = 556, 6.3%), communication & monitoring needs (*n* = 189, 2.1%), and reliability needs (*n* = 158, 1.8%). Each main category was represented across multiple products and product types: product coverage ranged from 27 to 61 of the 64 products in the focused corpus, and coverage across the 22 age-friendly product categories ranged from 13 to 21 categories (Supplementary Table 7).

Because reviews could express more than one need, total coding occurrences exceeded mutually exclusive primary-category frequencies. The most frequent main-category co-occurrences were interaction needs with service assurance needs (*n* = 912), care support needs with service assurance needs (*n* = 683), care support needs with interaction needs (*n* = 654), quality needs with interaction needs (*n* = 579), and interaction needs with value needs (*n* = 504). These co-occurrences indicate overlapping concerns within individual reviews and are not interpreted as causal or sequential relationships. Complete basic-category frequencies, the main-category co-occurrence matrix, and distributions across the 22 product categories are provided in Supplementary Tables 8–10.

Reviews expressing multiple needs were assigned both primary and secondary codes rather than being reduced to a single conceptual description. The primary main category represented the dominant evaluative focus of the review, while other explicitly expressed needs were retained as secondary codes. For example, one wheelchair review discussed portability, customer service, ease of use, safety, and value. Value needs were assigned as the primary category because comparative value constituted the central evaluation, while the other concerns were preserved as secondary codes. Another review emphasized both effort-saving transfer functions and ease of operation; it was therefore assigned primarily to care support needs, with interaction needs retained as a secondary category. Re-examination of these multi-need reviews confirmed that their expressed concerns could be represented by the existing code definitions, and no conceptually distinct category emerged.

## 5 Model explanation

The category explanations below distinguish three sources of evidence: experiences explicitly reported in the reviews, interpretations developed through coding, and contextual findings from previous studies. Review excerpts are used to identify expressed needs, while external literature is used to interpret their broader relevance. The review analysis itself does not constitute an evaluation of clinical or behavioral outcomes.

### 5.1 Quality needs

Quality needs can be categorized into four basic categories: noise control, packaging quality, portable design, and material craftsmanship. Together, these categories describe evaluations of the product’s sensory, material, handling, and spatial qualities. Although some of these attributes also apply to general consumer products, their relevance in age-friendly contexts is shaped by comfort requirements, mobility limitations, care environments, and continued product use.

Noise control refers to a product’s acoustic performance during operation and its perceived influence on the usage environment. One reviewer noted, “The main unit has a loud voice output, but the volume is adjustable. At night, it can be lowered to facilitate rest.” This account directly identifies adjustable volume as a need in quiet and nighttime settings. Previous research associating prolonged noise exposure with sleep disturbance and anxiety (55) provides broader context for the relevance of acoustic control, particularly in nighttime and rest settings.

Packaging quality encompasses protective performance, visual presentation, and the usability of the unboxing process. One reviewer commented, “SF Express delivered the item quickly, and the packaging was exquisite—it even had a sliding lid, which was quite interesting.” This statement reports a favorable initial impression of both delivery and packaging design. We interpret such accounts as indicating that protection, presentation, and intuitive opening mechanisms contribute to the perceived quality of age-friendly products.

Portable design reveals a product’s ability to support mobility across different contexts, including considerations of size, weight, and foldability. Reviews suggest that portability directly influences usability in daily life. As one user noted, “The product is of good quality, lightweight, and can be folded to fit easily into a car trunk.” Such features facilitate transportation and expand the range of use scenarios, thereby improving convenience and supporting more active lifestyles among older users.

Material craftsmanship relates to the tactile, functional, and esthetic qualities of a product. The comment “It looks stylish, fits well, and the workmanship and materials are excellent” expresses a favorable evaluation of appearance, fit, workmanship, and materials. We interpret these accounts as showing that perceived comfort, durability, and reduced medicalized appearance are relevant components of product experience and dignity in age-friendly contexts.

### 5.2 Service assurance needs

Service assurance needs reflect expectations concerning external support throughout product acquisition, deployment, use, and maintenance. These needs can be further classified into four basic categories: ease of installation, service reliability, customer service professionalism, and logistics efficiency. They describe vendor- and service-related conditions rather than intrinsic product functions, but remain relevant because complex age-friendly products may require timely delivery, professional configuration, and continued assistance to support safe and effective use.

Ease of installation refers to the perceived simplicity of moving from product delivery to operational use. The statement “The wood grain is clear and the color is accurate, and the product features easy installation” directly expresses a preference for low-complexity setup. The Technology Acceptance Model identifies perceived ease of use as an important influence on technology acceptance (56), providing theoretical context for interpreting installation simplicity as a potential usage barrier or facilitator.

Service reliability emphasizes the consistency and dependability attributed to service providers over time. One reviewer stated, “We’ve purchased products from them for several clinics in our family; the service is excellent, the products are reliable, and we have never encountered any issues.” The account combines repeated purchasing with a favorable evaluation of service reliability and therefore illustrates how dependable service was associated with trust in this specific review context.

Customer service professionalism encompasses the technical competence, communication approach, and problem-solving support attributed to service personnel. One reviewer noted, “Although it’s very simple, customer service patiently explained the logic to me. There were no false alarms or missed alarms.” This account highlights patient explanation and technical guidance as valued aspects of customer service and suggests that professional support can reduce uncertainty during product configuration and use.

Logistics efficiency reflects reviewers’ evaluations of delivery timeliness and completeness. The comment “The logistics and goods were both excellent” directly reports a favorable delivery experience. We interpret timely and complete delivery as a relevant service need, particularly when products are purchased for immediate home-care situations.

### 5.3 Reliability needs

Reliability needs encompass four basic categories: power performance, battery life, measurement accuracy, and data integration. Together, these categories reflect users’ expectations regarding product stability, accuracy, and durability.

Power performance refers to the intensity and precision of a product’s output during physical operation. User reviews indicate that performance quality directly affects perceived effectiveness. For example, one user noted that “the 6-core motor delivers distinct layers of percussion…with ultra-precise targeting of back acupoints.” Such performance not only enhances physical comfort but also reinforces users’ perception of effective intervention.

Battery life describes how long a product can operate without external power. One reviewer stated, “After a period of use, the older user is very satisfied. The product has good quality, long battery life.” This comment directly associates longer battery duration with a favorable usage evaluation. We therefore interpret battery life as a need related to charging convenience and continuity of use.

Measurement accuracy reflects reviewers’ expectations that device-generated physiological data should be consistent and dependable. Statements such as “The ECG results match those from physical exams” and “The heart rate matches my self-measurements” illustrate how reviewers evaluated perceived consistency by comparing device outputs with other available measurements. These accounts identify measurement accuracy and data reliability as important user-expressed needs.

Data integration emphasizes a device’s ability to support cross-platform communication, remote data sharing, and continuous data management. User feedback indicates that integration capabilities extend the functional value of monitoring devices. As one user noted, “Data can be shared remotely with the attending physician, eliminating the need for older adult to make frequent trips to the hospital.” By transforming fragmented home monitoring into continuous health records, data integration facilitates coordination between home care and formal healthcare services, thereby improving access to medical resources.

### 5.4 Care support needs

Care support needs refer to evaluations of a product’s practicality in supporting daily activities such as toileting, repositioning, and personal hygiene. These needs concern both the older adult receiving assistance and the family or professional caregiver providing it. They can be categorized into four basic categories: effort-saving effectiveness, care convenience, mattress comfort, and functional integration.

Effort-saving effectiveness reveals the extent to which a device reduces physical strain when assisting or replacing human labor in demanding care tasks. User feedback indicates that such features are closely related to both user comfort and caregiver burden. For example, one user noted, “It’s not only more comfortable than having someone assist, but it also saves a lot of effort…moving to the living room or bathroom is very convenient.” Given that family caregivers often face occupational health risks such as lower back injuries (57), effort-saving designs can reduce physical strain on caregivers while enhancing the older user’s sense of safety during movement.

Care convenience emphasizes a product’s ability to simplify specific care procedures, such as toileting, standing, and transferring. User comments suggest that improved convenience can significantly reduce dependence on multiple caregivers. For instance, one user stated, “It solves the problem of older adult having difficulty getting up and moving to the toilet; one person can provide care with ease.” This indicates that enhanced convenience enables tasks that previously required coordinated assistance to be performed more independently, thereby improving care efficiency.

Mattress comfort reflects perceived support, softness, and contact-surface comfort. Medical evidence identifies inappropriate mattress conditions as a risk factor for pressure ulcers among older adults with limited mobility (58). In the review corpus, however, users primarily emphasized subjective comfort; for example, one reviewer noted, “The mattress used to be thin and hard.but now it feels much softer when lying on it.” The review evidence therefore supports mattress comfort as an expressed need, while the cited medical literature provides the broader clinical context.

Functional integration describes the degree of coordination among different care modules within a product. User feedback indicates a strong demand for integrated care solutions. For example, suggestions such as “integrating the perineal cleansing function into the bed system itself and enabling operation via a built-in remote control” reflect users’ preference for more systematic care configurations. Higher levels of integration allow multiple functions to be controlled through a unified interface, which can reduce operational complexity, lower cognitive burden, and support more efficient and continuous care processes.

### 5.5 Value needs

Value needs refer to evaluations of the economic and emotional value associated with age-friendly products. Price competitiveness reflects the relationship between acquisition cost, functionality, and quality, whereas psychological security reflects the reassurance and reduced worry associated with product acquisition and use. These evaluations may be expressed by older users, family purchasers, or caregivers.

Price competitiveness reflects users’ assessment of whether a product delivers adequate value relative to its cost. User feedback indicates that affordability, when combined with satisfactory quality, is a key factor in perceived value. For example, one user commented, “The quality is excellent. More importantly, the price is affordable,” illustrating a positive evaluation of cost-effectiveness. Competitive pricing can lower barriers to adoption and help reduce the financial burden associated with long-term care, thereby enabling broader access to age-friendly products.

Psychological security emphasizes the intangible value that products provide by reducing perceived risks and alleviating anxiety. User comments suggest that such value extends beyond functional benefits to influence family well-being. As one user noted, “When parents are happy and at ease, their children can also experience a stronger sense of security. Spending a few thousand yuan on this garment is like buying insurance for your parents—if they were to fall, the cost would far exceed a few thousand yuan.” By offering preventive protection, such as fall prevention, age-friendly products can transform uncertain health risks into more manageable conditions, thereby enhancing users’ sense of security and supporting family peace of mind.

### 5.6 Interaction needs

Interaction needs encompass four basic categories: ease of use, measurement convenience, data visualization, and reporting functionality. These categories concern the accessibility and usability of product operation, feedback, and information processing for older users as well as caregivers or purchasers who assist with product configuration and use.

Ease of use refers to the intuitiveness of a product’s interaction logic and its associated learning curve. User feedback indicates that intuitive operation is essential for reducing barriers to adoption. For example, one user noted, “Even if you do not understand electronics, this is still very easy to operate and simple to get the hang of,” highlighting users’ reliance on intuitive interaction. By simplifying operational steps and incorporating features such as voice input, age-friendly products can enhance older users’ sense of self-efficacy and reduce resistance to new technologies.

Measurement convenience highlights the smoothness and simplicity of the physiological data collection process. User feedback suggests that complex procedures can significantly undermine usability. For instance, one user reported, “I bought this to take an ECG for my older adult when they were not feeling well, but the operation was so cumbersome and complicated that my parent could not figure it out at all. Even I, as a young person, found it complex—the results of the three-lead measurement could only be viewed on a computer.” This example illustrates how process complexity negatively affects user experience. In home settings, age-friendly products should adopt simplified operational logic, minimizing complex wiring and reducing reliance on specialized devices.

Data visualization reflects the perceived clarity and accessibility of information presentation. For example, one user noted, “It comes with a hallway display screen. When the bedside unit makes a call, the main unit announces the room and bed number, and the hallway display screen also announces the room and bed number—i’s a very good set of equipment,” demonstrating the value of multimodal feedback. We interpret combinations of visual and voice output as design features that may make information more accessible across different usage contexts.

Reporting functionality concerns the ability to convert device data into structured and shareable outputs. One reviewer stated, “Reports can be exported and printed at any time—I can take them straight to the hospital and use them immediately; it’s so convenient.” This comment directly reports perceived convenience in preparing information for communication with healthcare providers and supports reporting functionality as an expressed need.

### 5.7 Communication and monitoring needs

Communication & monitoring needs reflect expectations regarding the stability of information transmission, the sensitivity of alert mechanisms, and coordination across multiple users and devices. These needs can be categorized into four basic categories: communication quality, call responsiveness, remote monitoring, and multi-device synchronization. In age-friendly contexts, they support communication between older product users and family or professional caregivers across spatial boundaries.

Communication quality denotes the stability and reliability of signal transmission in complex environments. User feedback indicates that stable communication is essential for ensuring the timely delivery of alerts. For example, one user noted, “The signal quality is excellent. There are three buildings, each with seven floors, and the main unit is placed in the first building, yet full signal coverage has been achieved. After 1 week of use, the signal has remained very stable, and the system also supports mobile phone notifications. Overall, it is a highly reliable system.” This suggests that high communication quality can maintain consistent system performance and prevent information loss due to network fluctuations.

Call responsiveness encompasses the system’s sensitivity in triggering alerts, the clarity of voice transmission, and the timeliness of feedback. User reviews indicate that responsiveness directly affects the effectiveness of emergency communication. For instance, one user commented, “The call is very sensitive, the voice is clear, and it is not affected by the surrounding environment,” highlighting the importance of reliable and immediate response mechanisms.

Remote monitoring enables caregivers to access real-time information about the care recipient’s condition and environment through networked devices. User feedback suggests that this function supports continuous care without requiring physical presence. For example, one user noted, “After connecting it to the network, real-time remote access is possible, and alerts are delivered promptly.” For family members who do not co-reside with older adults, remote monitoring provides a non-intrusive means of maintaining awareness and reassurance.

Multi-device synchronization highlights the coordinated delivery of alerts and information across multiple devices and users. User feedback indicates that such coordination enhances collective response capacity. For example, one user stated, “The older adult has difficulty moving around at home, we are busy with work, and they do not know how to use a smartphone. We bought this call device for them, which can connect to five phones simultaneously. I had my wife, children, and the neighbor all register their numbers, so whoever is closest can respond immediately.” This example illustrates a collaborative care mechanism based on a distributed care network (59), enabling timely intervention through shared responsibility.

### 5.8 Safety needs

Safety needs reflect users’ expectations regarding the prevention of accidental injuries, the provision of physical protection, and the stability of dynamic operations. This main category can be divided into two basic categories: safety protection and safety braking. In age-friendly contexts, safety design has evolved from passive risk avoidance to active intervention, aiming to provide reliable and proactive protection for older adults through the integration of intelligent sensing and physical structures.

Safety protection focuses on the product’s ability to prevent injuries and mitigate harm in high-risk situations. User feedback indicates that smart protective devices can effectively reduce the severity of accidents. For example, some users reported positive experiences with smart anti-fall clothing, stating, “When the older adult fell a few days ago, the airbag deployed and prevented injury.” Falls are a major risk factor for disability among older adults, and wearable devices equipped with early warning and cushioning functions can reduce impact forces and lower the likelihood of severe injuries such as fractures. This form of “flexible protection” not only provides physical protection but also reinforces users’ confidence and psychological sense of security through successful risk mitigation experiences.

Safety braking addresses perceived stability and control during product movement. One reviewer described a smart wheelchair as “a bit heavy, but very stable.the intelligent brakes are very useful, so there is no need to worry about slipping when going uphill or downhill.” This account directly reports perceived braking stability and reassurance, supporting reliable braking as a safety-related design need.

### 5.9 Four-layer conceptual framework of user needs concerning age-friendly products

**Figure 3:**
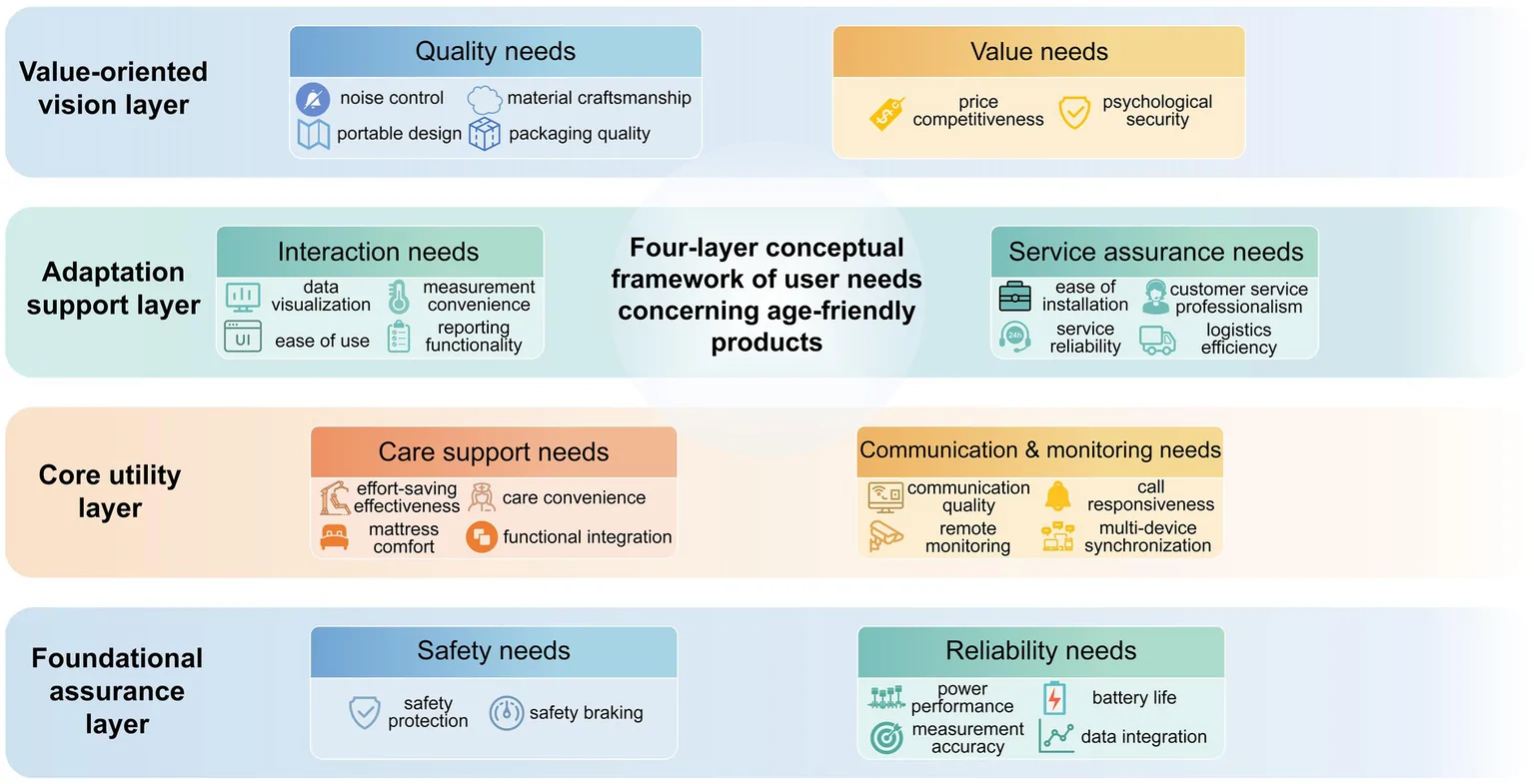
Four-layer conceptual framework of user needs concerning age-friendly products.

This study identifies 8 main categories and 28 basic categories of user needs derived from consumer reviews concerning age-friendly products and organizes them into a four-layer conceptual framework comprising the foundational assurance layer, core utility layer, adaptation support layer, and value-oriented vision layer (Figure 3).

The foundational assurance layer comprises safety and reliability needs, emphasizing essential safeguards in areas such as safety protection, measurement accuracy, data integration, and battery life. Existing research supports the importance of these concerns, indicating that older users’ willingness to adopt age-friendly products is substantially influenced by their perceptions of safety and technological risk (60). This consideration is particularly important because age-friendly products are not merely general consumer smart devices but are closely associated with physical safety, health management, and daily care. Consequently, significant deficiencies in safety or reliability may reduce user trust and hinder the integration of these products into real-life contexts.

The core utility layer captures the functional value of age-friendly products by addressing care support needs and communication & monitoring needs, including effort-saving effectiveness, care convenience, functional integration, communication quality, remote monitoring, call responsiveness, and multi-device synchronization. It focuses on whether products can effectively operate within everyday life and health management contexts, particularly in scenarios involving care, monitoring, communication, and timely response. This functional orientation is consistent with prior research identifying medical care and daily living support as central functions of smart senior care (61, 62). Within the proposed framework, these needs represent the practical utility through which age-friendly products support users and caregivers in everyday contexts.

The adaptation support layer concerns how product features are translated into usable and comprehensible experiences. It encompasses interaction needs and service assurance needs: the former relate to ease of operation, measurement convenience, data visualization, and reporting functionality, while the latter involve ease of installation, service reliability, customer service professionalism, and logistics efficiency. Despite advances in functional design, many age-friendly products exhibit notable deficiencies in interface interaction, usage guidance, and after-sales support. Consequently, even products with clear functional value may not achieve effective usability, contributing to the “high demands but low utilization” paradox among older adults (63). Although prior studies have primarily attributed this phenomenon to digital literacy and general usability constraints (64), the proposed framework further highlights the combined role of interaction design and service support in connecting product functions with effective use.

The value-oriented vision layer comprises quality needs and value needs, capturing users’ evaluations of product experience, psychological security, and long-term value. Quality needs pertain to concrete experiential attributes such as material craftsmanship, packaging quality, and noise control, whereas value needs include price competitiveness and psychological security. Prior research indicates that perceptions of product quality, emotional attachment, and value cognition significantly shape older adults’ willingness to continue using age-friendly products (60). At a broader level, policy and sustainable development perspectives emphasize that smart senior care should contribute to quality of life, independence, and overall well-being among older adults (65). By incorporating quality and value into the conceptual framework, the study broadens attention from product functionality and usability to high-quality experiences and long-term value recognition, including whether products are perceived as usable, useful, easy to use, desirable, and worth continuing to use.

In summary, the proposed framework organizes review-derived needs into four interrelated layers. The foundational assurance layer addresses safety and reliability; the core utility layer represents care, monitoring, and communication functions; the adaptation support layer concerns interaction and service conditions that facilitate effective use; the value-oriented vision layer captures quality evaluation, psychological security, and perceived value. These layers provide complementary perspectives on age-friendly product needs and may interact across different products, users, and usage contexts. Unlike approaches that present user needs as isolated factors, the four-layer conceptual framework offers an integrated structure for understanding how assurance, utility, adaptation support, and value evaluation are connected in the use of age-friendly products.

The framework encompasses needs related to product use, caregiving, purchasing, and service provision. These categories were distinguished according to the object and context of the expressed need. Although some categories also occur in general consumer settings, their significance in this framework lies in their recurring relevance to the acquisition and use of age-friendly products, particularly in contexts involving functional limitations, care dependence, and safety-sensitive use.

## 6 Discussion and conclusion

### 6.1 Theoretical contributions

This study seeks to advance understanding of how user needs concerning age-friendly products can be structurally organized in real-world product contexts. With the rapid development of the smart senior care industry, scholarly attention has increasingly focused on such needs. However, existing research has largely concentrated on identifying individual need factors, product design principles, and technology acceptance, while offering limited insight into the structural organization of different types of needs (22, 24, 60). Moreover, most prior studies rely on questionnaires or interviews, which may be limited in capturing naturally occurring expressions embedded in product acquisition, caregiving, use, and service encounters (4, 17). To address these limitations, this study employs computational grounded theory to analyze large-scale consumer reviews and identify user needs expressed in real-world product contexts. Building on this analysis, we develop a four-layer conceptual framework that distinguishes the roles of assurance, practical utility, adaptation support, and value evaluation. In doing so, the study provides a more comprehensive, integrated, and context-sensitive perspective on user needs concerning age-friendly products. More specifically, it makes three theoretical contributions to age-friendly product research.

First, we develop a four-layer conceptual framework that distinguishes four different but complementary roles of needs within age-friendly product contexts. Existing research has largely treated user needs as independent factors or dimensions, offering limited insight into how they can be organized within an integrated conceptual structure (60, 61, 65). Drawing on large-scale user-generated data, the foundational assurance layer represents requirements for safe, stable, and dependable product use; the core utility layer captures the practical functions provided for care, monitoring, communication, and daily activities; the adaptation support layer represents the interaction and service conditions that make product functions practically accessible; and the value-oriented vision layer captures evaluations of product quality, economic value, and psychological value. This organization moves beyond a flat aggregation of discrete product attributes by integrating product functions, interaction conditions, caregiving contexts, service provision, and value judgments within a coherent conceptual architecture. In particular, identifying interaction and service support as a shared adaptation role provides a structural perspective on the “high demands but low utilization” phenomenon observed in practice (63).

Compared with the proposed framework, recent reviews of gerontechnology and emerging technologies primarily explain technology acceptance through interacting personal, technological, environmental, and social factors (66, 67). Research on digital-health implementation and assistive-technology adoption further emphasizes stakeholder and caregiver involvement, organizational and service contexts, usability, cost, privacy, training, and user-centered design (68, 69). The present framework complements these acceptance-, adoption-, and implementation-oriented perspectives by focusing on the conceptual roles performed by needs expressed across product acquisition, everyday use, caregiving, and service encounters. Its contribution therefore lies not in treating safety, usability, reliability, service, or value as previously unidentified needs, but in integrating these dispersed concerns into a four-layer conceptual architecture that clarifies their different roles within age-friendly product contexts.

Second, we broaden the data sources and contextual scope of user needs research on age-friendly products by leveraging user-generated content (UGC). Although prior studies have increasingly examined user needs in this domain, they have predominantly relied on traditional methods such as questionnaires and interviews, which may be constrained by sample size and elicited or self-reported responses (70, 71). In contrast, online review data offers large-scale, continuously updated, and contextually embedded insights into users’ experiences, enabling a more comprehensive understanding of how user needs are expressed and shaped in actual usage scenarios (72, 73). Accordingly, we analyze consumer reviews from e-commerce platforms to capture context-specific expressions of user needs across purchasing, caregiving, product use, and service encounters. The findings show how UGC can complement conventional data sources by revealing recurrent patterns of need expression and experience that may receive less attention in questionnaires and interviews. In doing so, this study complements existing user needs research with a large-scale, naturally occurring source of data and advances a more context-sensitive understanding of how user needs are expressed in age-friendly product contexts.

Third, we contribute to the methodological advancement of user needs research on age-friendly products by applying computational grounded theory to large-scale user-generated data. By integrating topic modeling with grounded coding, this approach enables systematic analysis and theory development from complex textual data while preserving the interpretive depth of qualitative inquiry. Although computational grounded theory has been applied in social media and online community research, its potential for analyzing complex user needs remains underexplored (10, 44). To bridge this gap, we adopt the structured analytical pathway of “pattern detection-pattern refinement-pattern confirmation” to construct a four-layer conceptual framework of needs associated with age-friendly products. This approach improves the scale and efficiency of data processing while supporting systematic conceptual development (74), thus providing a feasible methodological foundation for identifying, organizing, and interpreting complex need structures in large-scale review corpora.

### 6.2 Practical implications

Building on the four-layer conceptual framework of user needs derived from consumer reviews, this study derives practical implications for four key stakeholders: users, product developers, e-commerce platforms, and the government.

From the user perspective, older adults and their family members should evaluate age-friendly products using a comprehensive needs-based framework rather than focusing solely on core functionalities. While core functions, such as care support and communication & monitoring, are central to product selection, safety and reliability serve as fundamental prerequisites that determine whether these functions can be effectively and sustainably utilized. Moreover, adaptation support factors including service assurance and user experience further shape actual usage experiences. Accordingly, users are encouraged to adopt a multidimensional evaluation approach that simultaneously considers functional performance, safety, reliability, and service support, thus reducing technological risks and improving usage effectiveness. Because the sampled reviews may include older users, caregivers, purchasers, and other consumers, these considerations should support, rather than replace, direct consultation with the older person who will use the product.

From the product developers’ perspective, the framework highlights four complementary areas for age-friendly product design. Within this structure, safety and reliability constitute the foundation for product use, whereas care support needs and communication & monitoring needs define the core utility of the product. Accordingly, product developers should first ensure compliance with fundamental needs and subsequently strengthen core functionalities such as health monitoring, remote care, and multi-device synchronization. On this basis, greater emphasis should be placed on the adaptation support layer by improving usability through optimized human–machine interfaces and streamlined operational processes, alongside enhanced logistics efficiency and professional service assurance. These improvements reduce the learning burden and operational complexity for older users, thereby facilitating smoother product use and higher levels of technology acceptance. Furthermore, product developers should improve product quality by optimizing materials and manufacturing processes, reducing device noise, and enhancing portability, so as to meet the quality expectations represented in the review corpus. In parallel, firms should strengthen users’ sense of psychological security and value recognition through well-defined product positioning and pricing strategies, thereby improving overall product competitiveness. Because reviewer roles could not be reliably distinguished in the present corpus, these implications should be treated as review-derived design priorities and further examined through participatory research involving identifiable older users and caregivers.

From the perspective of e-commerce platforms, online reviews can be systematically analyzed to identify user needs and support product optimization and market decision-making. As a form of user-generated content, reviews capture consumer experiences and expressed needs in product-related contexts and have become an important source for understanding user concerns (75). Platforms can use text-mining techniques to transform large-scale review data into structured insights for product improvement. They can also develop evaluation systems for age-friendly products that incorporate dimensions such as safety, reliability, usability, and service support, helping consumers identify suitable products more efficiently and reducing information-search costs.

From the governmental perspective, advancing the age-friendly industrial ecosystem requires coordinated policy support and effective governance. As population aging accelerates, smart senior care and age-friendly products are becoming increasingly important to the digital economy and care provision. Governments can promote the sector by establishing technical and service standards, supporting technological innovation, and strengthening quality supervision (62, 76). The needs represented in the review corpus can inform these efforts by highlighting concerns related to safety, reliability, usability, care functions, and service support. Industry-academia-research collaboration and demonstration projects in community care settings can further support responsible development (77–79), while digital literacy training, community-based technical assistance, and accessible public services can promote digital inclusion (80). Together, these measures can support more inclusive and responsible development of age-friendly products and services.

### 6.3 Limitations and future research

This study has sought to develop a four-layer conceptual framework of user needs for age-friendly products using online review data and computational grounded theory. The framework contributes to understanding how different types of needs can be organized according to the complementary roles of assurance, practical utility, adaptation support, and value evaluation, while offering practical guidance for product and service development. Despite these contributions, our study has several limitations that provide opportunities for further research.

First, the reviewer population and the representativeness of the corpus were constrained by the characteristics of platform-based data. Taobao and Tmall did not consistently disclose reviewers’ age, relationship to the product user, or basis of experience, so the corpus may combine the perspectives of older users, family purchasers, caregivers, institutional staff, and other consumers. Access to e-commerce and voluntary review posting are also uneven: older adults affected by the digital divide, cognitive impairment, limited internet access, or limited family support may be underrepresented, while posted reviews may be influenced by unusually positive or negative experiences, incentives, platform moderation, and social desirability. In addition, the observed framework may partly reflect the product composition, transaction patterns, platform visibility, and review-posting practices of the sampled marketplaces rather than a universal structure of needs across all age-friendly products and populations. The findings should therefore be interpreted as user needs derived from consumer reviews within the sampled Chinese e-commerce context. Future studies could examine the framework across different platforms, sociocultural settings, and care systems. Review data could also be triangulated with direct research involving clearly identified older users and caregivers.

Second, this study focuses on the cross-product conceptual organization of user needs rather than conducting comparative analyses of individual product categories. Given the substantial variation in product functions and usage contexts, the relative salience and specific manifestations of needs may differ across product categories. Future research should examine such differences to determine how the proposed framework is expressed and refined within particular product contexts. Such analyses would provide a more fine-grained understanding of product-specific needs while extending the cross-product conceptual organization developed in this study.

Finally, this study employs a computational grounded theory framework that integrates deep learning-based text mining techniques with grounded theory coding procedures. This integration improves the efficiency of large-scale text analysis while preserving the interpretive strength of qualitative research. However, our analysis is restricted to cross-sectional review data and does not capture the dynamic evolution mechanisms of user needs over time. Future research could extend this work by incorporating longitudinal designs or time-series review data to examine how user needs evolve over time, particularly in response to product iteration and technological change, thus shedding further light on the evolutionary mechanisms underlying user needs for age-friendly products.

## Data Availability

The datasets presented in this article are not readily available because the data analyzed in this study is subject to the following licenses/restrictions: the raw review data are subject to platform terms, copyright restrictions, and privacy considerations and therefore cannot be publicly redistributed. De-identified derived data, aggregated results, and relevant analytical materials may be made available by the corresponding author upon reasonable request, subject to applicable platform conditions, legal requirements, and institutional approval. Requests to access the datasets should be directed to leezz18@163.com.
