# Supplementary material for: Consumer-expressed needs concerning age-friendly products in China: a computational grounded theory analysis of online reviews
Source: Front Public Health. 2026 Sep 9;14:1869463. doi: 10.3389/fpubh.2026.1869463 (PMC13597768; doi:10.3389/fpubh.2026.1869463)
Supplement: Supplementary file 1 [file Table_1.DOCX]

Supplementary Material

**Table 1. Examples of open coding**

| **Keywords** | **Representative texts** | **initial concepts** | **basic categories** |
| --- | --- | --- | --- |
| mobile phone | The signal quality is excellent. There are three buildings, each with seven floors, and the main unit is placed in the first building, yet full signal coverage has been achieved. After one week of use, the signal has remained very stable, and the system also supports mobile phone notifications. Overall, it is a highly reliable system. | signal coverage | communication quality |
| easy installation | The wood grain is clear and the color is accurate, and the product features easy installation. We first saw the product at the seller’s exhibition and then customized a batch directly from the factory. The quality is excellent, and we received a wholesale price. The leadership team was very satisfied. | easy installation | ease of installation |
| position transfer | Since purchasing this device, position transfer for the older adult has become much easier. It is very comfortable to use and, when combined with the bed, is not only more comfortable than manual assistance but also significantly reduces physical effort. It allows convenient position transfer to the living room or bathroom and offers high value. | effort-saving position transfer | effort-saving effectiveness |
| price | Positive feedback! The product has been received, and the quality is excellent. More importantly, the price is affordable. | cost-effectiveness | price competitiveness |
| customer service | Placed under the bed sheet for the older adult, it has reduced concerns during the night compared to before. An alarm is triggered when the user sits up. The customer service attitude is excellent. I initially placed it incorrectly. Although it’s very simple, customer service patiently explained the logic to me. After adjustment, the system worked perfectly. After several days of use, there were no false alarms or missed alerts. | patient customer service | customer service professionalism |
| voice | The main unit has a loud voice output, but the volume is adjustable. At night, it can be lowered to facilitate rest, while during the day, it can be increased when more people are present without causing issues. | adjustable voice output | noise control |
| daily care | The device significantly improves efficiency in daily care, making it less time-consuming and physically demanding to care for the older adult. | reduced care burden | care convenience |
| battery life | After a period of use, the older user is very satisfied. The product has good quality, long battery life, and convenient features such as one-touch dialing and automatic call answering, which enhance usability. | long battery life | battery life |
| heart rate | After testing before reviewing. The ECG results match those from physical exams and the heart rate matches my self-measurements. It is impressive that such a compact device can maintain this level of performance. | accurate heart rate measurement | measurement accuracy |
| measurement | I bought this to take an ECG for my older adult when they weren’t feeling well, but the operation was so cumbersome and complicated that my parent couldn’t figure it out at all. Even I, as a young person, found it complex—the results of the three-lead measurement could only be viewed on a computer, and alternative measurement methods (palm or ankle contact) are inaccurate. The product does not match its description and creates unnecessary frustration. | complex measurement process | measurement convenience |
| service | After my father was diagnosed with coronary heart disease, a cardiologist at a top-tier hospital recommended this device. It allows real-time monitoring at home. The operation is relatively simple and easy to understand. Reports can be exported and printed at any time—I can take them straight to the hospital and use them immediately; it’s so convenient. The one-year free service is also highly valuable. | exportable reports | reporting functionality |
| remote access | Concerned about my mother’s health, a doctor recommended purchasing a monitoring device. The customer service suggested this model, which is suitable. After connecting it to the network, real-time remote access is possible, and alerts are delivered promptly. | remote access capability | remote monitoring |
| sense of security | The flagship version has excellent workmanship and durable materials. When parents are happy and at ease, their children can also experience a stronger sense of security. Spending a few thousand yuan on this garment is like buying insurance for your parents—if they were to fall, the cost would far exceed a few thousand yuan. | sense of security | psychological security |
| fold | The product is of good quality, lightweight, and can be folded to fit easily into a car trunk. The older user enjoys using it for outings. | foldable storage | portable design |
| airbag | This product deserves recognition. I initially purchased it with some skepticism, but when the older adult fell a few days ago, the airbag deployed and prevented injury. It is a trustworthy product. | airbag deployment | safety protection |
| downhill | The intelligent brakes are very useful, so there is no need to worry about slipping when going uphill or downhill, which significantly enhances safety. Initially, I practiced using the first gear. | automatic braking function | safety braking |

**Table 2. Results of axial coding**

| **Main Categories** | **Basic Categories** | **Definition of Basic Categories** |
| --- | --- | --- |
| quality needs | noise control | The noise level during product operation |
|  | packaging quality | The protective measures during transportation and the initial impression during unboxing |
|  | portable design | The control of product size and the convenience of storage, adapting to both home and outdoor scenarios |
|  | material craftsmanship | The product materials, workmanship details, and the tactile experience of user-contact surfaces |
| service assurance needs | ease of installation | The simplicity of the installation process and the availability of professional support |
|  | service reliability | The responsiveness of after-sales service and its ability to resolve problems |
|  | customer service professionalism | The professional competence of customer service personnel |
|  | logistics efficiency | The delivery speed and service details in the logistics process |
| reliability needs | power performance | The output capability and stability of the core power system |
|  | battery life | The duration of battery usage and the charging experience, determining continuity in daily use |
|  | measurement accuracy | The professional reliability of health monitoring data |
|  | data integration | The capability to exchange and manage data with external systems or devices |
| care support needs | effort-saving effectiveness | The extent to which the product reduces the physical burden of caregivers |
|  | care convenience | The practical design of daily care tasks for dependent older adults |
|  | mattress comfort | The impact of contact surface materials on the comfort of long-term bedridden older adults |
|  | functional integration | The degree and practicality of integrating multiple care functions within the product |
| value needs | price competitiveness | The comparative advantage of price relative to similar products and its alignment with functionality and quality |
|  | psychological security | The subjective emotional value provided by the product and its role in alleviating safety-related anxiety among older adults |
| interaction needs | ease of use | The user-friendliness of interface and interaction design for older users |
|  | measurement convenience | The simplicity of the health data collection process |
|  | data visualization | The intuitiveness and readability of health data presentation |
|  | reporting functionality | The capability of data export and report generation |
| communication & monitoring needs | communication quality | The stability and accuracy of the communication module |
|  | call responsiveness | The timeliness and accuracy of emergency call triggering and response |
|  | remote monitoring | The capability for remote viewing and intervention |
|  | multi-device synchronization | The ability to support multiple devices receiving information or being controlled simultaneously |
| safety needs | safety protection | The effectiveness of proactive protection mechanisms in mitigating risks such as falls and collisions |
|  | safety braking | The reliability and responsiveness of braking systems in mobility devices (wheelchairs, transfer devices) |

**Supplementary Table 1.** **Keywords and grouping rules of Chinese search combinations**

| **Groups** | **Keywords** | **Translation in Chinese** |
| --- | --- | --- |
| 1 | 适老化，智慧养老，智能养老， 智慧化养老，智能化养老，助老，康养 | age-friendly, smart senior care, intelligent older adults care, also referring to smart senior care, also referring to intelligent older adults care, older adults assistance, health care |
| 2 | 产品，设备，商品，物品，设施 | product, device, commodity, item, facility |

The search queries were formed by combining each keyword from the first group with each keyword from the second group, resulting in pairs such as “age-friendly product” “age-friendly device” “smart senior care commodity”.

**Supplementary Table 2. Distribution of products and raw reviews across age-friendly product categories**

| **No.** | **Age-friendly product category** | **Products** | **Raw reviews** |
| --- | --- | --- | --- |
| 1 | Age-friendly electric nursing beds | 2 | 3,964 |
| 2 | Rehabilitation training devices for older adults | 5 | 3,887 |
| 3 | Electric wheelchairs for older adults | 4 | 2,765 |
| 4 | Walking assistance devices for older adults | 1 | 949 |
| 5 | Personal emergency alert devices for older adults | 3 | 863 |
| 6 | Smart nurse-call and eldercare management systems | 5 | 151 |
| 7 | Toileting assistance devices for older adults | 4 | 857 |
| 8 | Age-friendly bathroom fixtures | 1 | 5 |
| 9 | Wearable health-monitoring devices for older adults | 3 | 563 |
| 10 | Sleep and blood oxygen monitors for older adults | 1 | 168 |
| 11 | Stair-climbing wheelchairs for older adults | 1 | 407 |
| 12 | Transfer assistance devices for older adults with mobility limitations | 8 | 1,232 |
| 13 | Wearable fall-protection devices for older adults | 5 | 503 |
| 14 | Airway-clearance assistance devices for older adults | 5 | 346 |
| 15 | Remote video communication devices for older adults | 3 | 195 |
| 16 | Companion robots for older adults | 1 | 40 |
| 17 | Bed-exit and fall-risk alarm devices for older adults | 4 | 98 |
| 18 | Residential stairlifts for older adults | 3 | 56 |
| 19 | Home ECG monitoring devices for older adults | 3 | 66 |
| 20 | Smart medication management devices for older adults | 1 | 8 |
| 21 | Vision-based fall-detection systems for older adults | 1 | 3 |
| 22 | Wearable mobility-assistance exoskeletons for older adults | 2 | 7 |
|  | **Total** | **66** | **17,133** |

**Supplementary Table 3. Complete BERTopic distribution and topic-selection decisions**

| **Topic** | **Reviews** | **%** | **Decision** | **Reason/role** |
| --- | --- | --- | --- | --- |
| -1 | 2,647 | 21.78 | Outlier | Reviewed in the low-frequency coverage check |
| 0 | 3,593 | 29.56 | Retained | Large, distinct, and interpretable thematic anchor |
| 1 | 1,354 | 11.14 | Retained | Large, distinct, and interpretable thematic anchor |
| 2 | 994 | 8.18 | Retained | Large, distinct, and interpretable thematic anchor |
| 3 | 845 | 6.95 | Retained | Large, distinct, and interpretable thematic anchor |
| 4 | 784 | 6.45 | Retained | Large, distinct, and interpretable thematic anchor |
| 5 | 261 | 2.15 | Retained | Large, distinct, and interpretable thematic anchor |
| 6 | 235 | 1.93 | Retained | Large, distinct, and interpretable thematic anchor |
| 7 | 201 | 1.65 | Not retained | Substantial overlap with Topic 1 |
| 8 | 171 | 1.41 | Not retained | Predominantly generic usage evaluations |
| 9 | 114 | 0.94 | Not retained | Insufficient semantic coherence |
| 10 | 109 | 0.90 | Not retained | Smaller topic; reviewed in the coverage check |
| 11 | 103 | 0.85 | Not retained | Smaller topic; reviewed in the coverage check |
| 12 | 101 | 0.83 | Not retained | Smaller topic; reviewed in the coverage check |
| 13 | 97 | 0.80 | Not retained | Smaller topic; reviewed in the coverage check |
| 14 | 93 | 0.77 | Not retained | Smaller topic; reviewed in the coverage check |
| 15 | 77 | 0.63 | Not retained | Smaller topic; reviewed in the coverage check |
| 16 | 72 | 0.59 | Not retained | Smaller topic; reviewed in the coverage check |
| 17 | 67 | 0.55 | Not retained | Smaller topic; reviewed in the coverage check |
| 18 | 63 | 0.52 | Not retained | Smaller topic; reviewed in the coverage check |
| 19 | 60 | 0.49 | Not retained | Smaller topic; reviewed in the coverage check |
| 20 | 57 | 0.47 | Not retained | Smaller topic; reviewed in the coverage check |
| 21 | 55 | 0.45 | Not retained | Smaller topic; reviewed in the coverage check |
| **Total** | **12,153** | **100.00** | **-** | **Complete BERTopic input corpus** |

**Supplementary Table 4. Original Chinese review excerpts and faithful English translations**

| **No.** | **Original Chinese excerpt** | **Faithful English translation** |
| --- | --- | --- |
| 1 | 主机声音是真大，不过可以调，晚上的时候可以调小点方便休息 | The main unit has a loud voice output, but the volume is adjustable. At night, it can be lowered to facilitate rest. |
| 2 | 货品顺丰发得也迅速，包装精美，还是滑盖式的，很有意思 | SF Express delivered the item quickly, and the packaging was exquisite - it even had a sliding lid, which was quite interesting. |
| 3 | 质量好，轻便，折叠后汽车后备箱完全可放下 | The product is of good quality, lightweight, and can be folded to fit easily into a car trunk. |
| 4 | 样式好看，上身效果不错，做工和材质都很好 | It looks stylish, fits well, and the workmanship and materials are excellent. |
| 5 | 板材纹路清晰颜色正，安装简单 | The wood grain is clear, the color is accurate, and the product is easy to install. |
| 6 | 我们家好几个诊所的都是在他家买的，服务很好，产品可靠，还没有出现过问题 | We have purchased products from this seller for several clinics in our family; the service is excellent, the products are reliable, and we have not encountered any problems. |
| 7 | 虽然很简单，但是客服还是给我耐心讲解逻辑……没有误报和漏报 | Although it was very simple, customer service patiently explained the logic to me...there were no false alarms or missed alarms. |
| 8 | 物流和货物都很好 | The logistics and the product were both excellent. |
| 9 | 6核动力捶打层次感明显……对应背部穴位超精准 | The six-core motor delivers distinct layers of percussion...with highly precise targeting of back acupoints. |
| 10 | 电量续航时间长，老人用一段时间下来挺满意 | The battery lasts a long time, and the older user has been quite satisfied after using it for a period of time. |
| 11 | 心电图和体检结果一样；心率和自测一样 | The ECG results match those from physical examinations, and the heart rate matches my self-measurements. |
| 12 | 可以远程分享数据给主治医生，不用老人频繁跑去医院 | Data can be shared remotely with the attending physician, eliminating the need for the older adult to make frequent trips to the hospital. |
| 13 | 不仅比人伺候起来舒服，更是省了不少力气……移动到客厅、厕所都非常方便 | It is not only more comfortable than having someone assist, but it also saves considerable effort...moving to the living room or bathroom is very convenient. |
| 14 | 解决了老人起床搬动上厕难的问题，一个人也可以轻松护理 | It solves the difficulty of helping the older adult get out of bed, move, and use the toilet; one person can provide care with ease. |
| 15 | 床垫很薄很硬……现在躺上去柔软多了 | The mattress used to be thin and hard...but now it feels much softer when lying on it. |
| 16 | 将私处冲洗功能集成到床系统本身和内嵌入遥控器操作 | The perineal cleansing function is integrated into the bed system itself and can be operated through a built-in remote control. |
| 17 | 质量非常的不错，关键是价格也不贵 | The quality is excellent. More importantly, the price is affordable. |
| 18 | 父母开心安心，子女就放心了 这衣服小几千块就相当于给父母买个保险，万一摔下来那就不是小几千块钱的问题了 | When parents are happy and at ease, their children can also feel reassured. Spending a few thousand yuan on this garment is like buying insurance for your parents - if they were to fall, the cost would far exceed a few thousand yuan. |
| 19 | 即使不懂电子产品，这个还是很好操作的，上手简单 | Even if you do not understand electronic products, this is still very easy to operate and simple to learn. |
| 20 | 为了给家里老人平时又不舒服的时候测一下心电图买的结果操作繁琐复杂老人家根本搞不懂，我一个年轻人都觉得复杂，三导联测量结果一定要在电脑上才能看到测量结果 | I bought this to take an ECG for my older adult when they were not feeling well, but the operation was so cumbersome and complicated that my parent could not figure it out at all. Even I, as a young person, found it complex - the results of the three-lead measurement could only be viewed on a computer. |
| 21 | 配了一个走廊显示屏，床头分机呼叫的时候，主机会播报多少房多少床，这个走廊显示屏也会播报多少房，多少床，很不错的一套设备 | It comes with a hallway display screen. When the bedside unit makes a call, the main unit announces the room and bed number, and the hallway display also announces the room and bed number. It is a very good set of equipment. |
| 22 | 报告可以随时导出，打印拿到医院就能用，太方便了 | Reports can be exported and printed at any time. I can take them directly to the hospital and use them immediately; it is very convenient. |
| 23 | 信号是真的好，3栋楼，每栋楼7层，主机放在第一栋的，都是实现了信号全覆盖，用了一周了，信号非常的稳定，而且还有手机提醒功能，很不错的一套系统 | The signal quality is excellent. There are three buildings, each with seven floors, and the main unit is placed in the first building, yet full signal coverage has been achieved. After one week of use, the signal has remained very stable, and the system also supports mobile phone notifications. Overall, it is a very good system. |
| 24 | 呼叫很灵敏，说话声音很清晰，没有受到周围环境的影响 | The call is very responsive, the voice is clear, and it is not affected by the surrounding environment. |
| 25 | 做了联网，能远程实时查看，而且报警很及时 | After connecting it to the network, real-time remote access is possible, and alerts are delivered promptly. |
| 26 | 老人在家行动不方便，我们又要上班忙，老人家又不会用手机，给他买了这个呼叫器，可以同时连接5个人的电话，我让我老婆孩子和隔壁邻居都绑定了电话，到时候谁离得近方便第一时间跑过去查看 | The older adult has difficulty moving around at home, we are busy with work, and they do not know how to use a smartphone. We bought this call device for them, which can connect to five phones simultaneously. I had my wife, children, and the neighbor all register their numbers, so whoever is closest can respond immediately. |
| 27 | 老人家前些天跌倒，衣服气囊弹开，老人家安然无恙 | When the older adult fell a few days ago, the garment’s airbag deployed, and the older adult was unharmed. |
| 28 | 重量一些，不过稳，我需要稳一点，这样不会侧翻等等；扶手可以打开，这点很实用，家人只能从侧面上车；智能刹车非常实用，上下坡不用担心溜坡，增加安全系数 | It is somewhat heavy, but stable. I need it to be stable so that it will not tip over. The armrest can be opened, which is very practical because my family member can only enter from the side. The intelligent brakes are very useful, so there is no need to worry about rolling when going uphill or downhill, which increases safety. |

Note: All English excerpts are faithful translations of the original Chinese reviews. Translation was assisted by DeepL Pro and independently checked by the fourth author against the Chinese originals.

**Supplementary Table 5. Product-grouped SVM classification performance by main category**

| **Main category** | **n** | **Precision** | **Recall** | **F1** |
| --- | --- | --- | --- | --- |
| Safety needs | 81 | 0.762 | 0.593 | 0.667 |
| Quality needs | 105 | 0.606 | 0.629 | 0.617 |
| Interaction needs | 95 | 0.592 | 0.611 | 0.601 |
| Value needs | 90 | 0.758 | 0.767 | 0.762 |
| Care support needs | 93 | 0.75 | 0.871 | 0.806 |
| Communication & monitoring needs | 77 | 0.944 | 0.883 | 0.913 |
| Service assurance needs | 99 | 0.689 | 0.737 | 0.712 |
| Reliability needs | 89 | 0.951 | 0.876 | 0.912 |
| Macro average | 729 | 0.756 | 0.746 | 0.749 |
| Weighted average | 729 | 0.748 | 0.742 | 0.743 |

**Supplementary Table 6. Confusion matrix for pooled outer-fold predictions**

| **Actual / predicted** | **SAF** | **QUA** | **INT** | **VAL** | **CAR** | **COM** | **SER** | **REL** |
| --- | --- | --- | --- | --- | --- | --- | --- | --- |
| **SAF** | 48 | 12 | 4 | 2 | 3 | 2 | 8 | 2 |
| **QUA** | 7 | 66 | 13 | 5 | 5 | 1 | 7 | 1 |
| **INT** | 2 | 15 | 58 | 5 | 7 | 0 | 7 | 1 |
| **VAL** | 2 | 4 | 4 | 69 | 3 | 1 | 7 | 0 |
| **CAR** | 0 | 2 | 4 | 4 | 81 | 0 | 2 | 0 |
| **COM** | 2 | 2 | 2 | 0 | 1 | 68 | 2 | 0 |
| **SER** | 2 | 4 | 8 | 5 | 7 | 0 | 73 | 0 |
| **REL** | 0 | 4 | 5 | 1 | 1 | 0 | 0 | 78 |

Note: Rows indicate coded categories and columns indicate predicted categories. SAF = safety; QUA = quality; INT = interaction; VAL = value; CAR = care support; COM = communication and monitoring; SER = service assurance; REL = reliability.

**Supplementary Table 7. Frequency and coverage of the eight main categories**

| **Main category** | **Primary n** | **%** | **Reviews coded as primary or secondary (n)** | **Products** | **Product categories** |
| --- | --- | --- | --- | --- | --- |
| service assurance needs | 2440 | 27.60% | 3593 | 61 | 21 |
| care support needs | 1876 | 21.20% | 2300 | 53 | 18 |
| interaction needs | 1361 | 15.40% | 2884 | 53 | 18 |
| value needs | 1213 | 13.70% | 1704 | 55 | 18 |
| quality needs | 1056 | 11.90% | 1761 | 50 | 19 |
| safety needs | 556 | 6.30% | 667 | 45 | 18 |
| communication & monitoring needs | 189 | 2.10% | 289 | 27 | 13 |
| reliability needs | 158 | 1.80% | 469 | 29 | 15 |

Note: Primary main-category frequencies sum to 8,849 because each review was assigned one primary main category. The counts of reviews coded to each main category as either primary or secondary are not mutually exclusive, because a multi-need review could be assigned to more than one main category.

**Supplementary Table 8. Frequency and coverage of the 28 basic categories**

| **Main category** | **Basic category** | **Reviews** | **%** | **Products** | **Product categories** |
| --- | --- | --- | --- | --- | --- |
| care support needs | care convenience | 1893 | 21.40% | 52 | 18 |
| care support needs | functional integration | 357 | 4.00% | 26 | 11 |
| care support needs | effort-saving effectiveness | 127 | 1.40% | 20 | 7 |
| care support needs | mattress comfort | 47 | 0.50% | 2 | 1 |
| communication & monitoring needs | communication quality | 174 | 2.00% | 24 | 13 |
| communication & monitoring needs | remote monitoring | 72 | 0.80% | 9 | 5 |
| communication & monitoring needs | call responsiveness | 53 | 0.60% | 14 | 8 |
| communication & monitoring needs | multi-device synchronization | 4 | 0.10% | 3 | 3 |
| interaction needs | ease of use | 2771 | 31.30% | 53 | 18 |
| interaction needs | measurement convenience | 108 | 1.20% | 6 | 5 |
| interaction needs | data visualization | 56 | 0.60% | 10 | 5 |
| interaction needs | reporting functionality | 5 | 0.10% | 4 | 3 |
| quality needs | material craftsmanship | 1566 | 17.70% | 46 | 18 |
| quality needs | portable design | 140 | 1.60% | 23 | 10 |
| quality needs | noise control | 58 | 0.70% | 6 | 6 |
| quality needs | packaging quality | 38 | 0.40% | 12 | 10 |
| reliability needs | power performance | 341 | 3.90% | 23 | 12 |
| reliability needs | battery life | 135 | 1.50% | 15 | 9 |
| reliability needs | measurement accuracy | 19 | 0.20% | 4 | 4 |
| reliability needs | data integration | 8 | 0.10% | 6 | 5 |
| safety needs | safety protection | 658 | 7.40% | 45 | 18 |
| safety needs | safety braking | 10 | 0.10% | 1 | 1 |
| service assurance needs | customer service professionalism | 2516 | 28.40% | 61 | 21 |
| service assurance needs | ease of installation | 1038 | 11.70% | 25 | 11 |
| service assurance needs | logistics efficiency | 710 | 8.00% | 34 | 14 |
| service assurance needs | service reliability | 120 | 1.40% | 14 | 10 |
| value needs | price competitiveness | 1495 | 16.90% | 47 | 18 |
| value needs | psychological security | 234 | 2.60% | 39 | 17 |

**Supplementary Table 9. Main-category co-occurrence matrix**

| **Category** | **CAR** | **COM** | **INT** | **QUA** | **REL** | **SAF** | **SER** | **VAL** |
| --- | --- | --- | --- | --- | --- | --- | --- | --- |
| CAR | 2300 | 21 | 654 | 269 | 55 | 69 | 683 | 254 |
| COM | 21 | 289 | 134 | 38 | 24 | 6 | 38 | 33 |
| INT | 654 | 134 | 2884 | 579 | 249 | 155 | 912 | 504 |
| QUA | 269 | 38 | 579 | 1761 | 127 | 88 | 452 | 273 |
| REL | 55 | 24 | 249 | 127 | 469 | 44 | 131 | 109 |
| SAF | 69 | 6 | 155 | 88 | 44 | 667 | 149 | 126 |
| SER | 683 | 38 | 912 | 452 | 131 | 149 | 3593 | 441 |
| VAL | 254 | 33 | 504 | 273 | 109 | 126 | 441 | 1704 |

Note: CAR = care support; COM = communication & monitoring; INT = interaction; QUA = quality; REL = reliability; SAF = safety; SER = service assurance; VAL = value. Diagonal cells show category coverage; off-diagonal cells show pairwise co-occurrence.

**Supplementary Table 10. Primary main category distribution across 22 age-friendly product categories**

| **Age-friendly product category** | **CAR** | **COM** | **INT** | **QUA** | **REL** | **SAF** | **SER** | **VAL** | **Total** |
| --- | --- | --- | --- | --- | --- | --- | --- | --- | --- |
| Age-friendly electric nursing beds | 1055 | 1 | 191 | 198 | 6 | 54 | 1043 | 237 | 2785 |
| Rehabilitation training devices for older adults | 414 | 3 | 248 | 223 | 11 | 43 | 363 | 266 | 1571 |
| Electric wheelchairs for older adults | 75 | 4 | 530 | 329 | 114 | 123 | 362 | 289 | 1826 |
| Walking assistance devices for older adults | 59 | 0 | 23 | 37 | 5 | 23 | 118 | 49 | 314 |
| Personal emergency alert devices for older adults | 37 | 15 | 74 | 72 | 6 | 22 | 88 | 66 | 380 |
| Smart nurse-call and eldercare management systems | 6 | 3 | 12 | 5 | 0 | 3 | 37 | 13 | 79 |
| Toileting assistance devices for older adults | 5 | 0 | 11 | 32 | 0 | 10 | 54 | 37 | 149 |
| Age-friendly bathroom fixtures | 0 | 0 | 0 | 0 | 0 | 0 | 1 | 0 | 1 |
| Wearable health-monitoring devices for older adults | 30 | 70 | 51 | 25 | 5 | 39 | 29 | 98 | 347 |
| Sleep and blood oxygen monitors for older adults | 7 | 22 | 7 | 2 | 1 | 7 | 36 | 17 | 99 |
| Stair-climbing wheelchairs for older adults | 17 | 0 | 56 | 14 | 4 | 2 | 55 | 23 | 171 |
| Transfer assistance devices for older adults with mobility limitations | 127 | 0 | 106 | 51 | 1 | 8 | 165 | 48 | 506 |
| Wearable fall-protection devices for older adults | 6 | 1 | 10 | 51 | 0 | 209 | 38 | 17 | 332 |
| Airway-clearance assistance devices for older adults | 10 | 1 | 6 | 6 | 4 | 1 | 15 | 16 | 59 |
| Remote video communication devices for older adults | 5 | 37 | 17 | 4 | 0 | 2 | 7 | 20 | 118 |
| Companion robots for older adults | 5 | 27 | 7 | 1 | 1 | 0 | 6 | 4 | 25 |
| Bed-exit and fall-risk alarm devices for older adults | 8 | 2 | 2 | 1 | 0 | 3 | 8 | 4 | 28 |
| Residential stairlifts for older adults | 7 | 0 | 7 | 4 | 0 | 5 | 8 | 6 | 37 |
| Home ECG monitoring devices for older adults | 3 | 3 | 3 | 0 | 0 | 1 | 5 | 3 | 18 |
| Smart medication management devices for older adults | 0 | 0 | 0 | 1 | 0 | 0 | 1 | 0 | 2 |
| Vision-based fall-detection systems for older adults | 0 | 0 | 0 | 0 | 0 | 1 | 0 | 0 | 1 |
| Wearable mobility-assistance exoskeletons for older adults | 0 | 0 | 0 | 0 | 0 | 0 | 1 | 0 | 1 |

Note: Counts represent primary main-category assignments and are provided to describe corpus coverage across product categories, rather than for between-category prevalence comparisons.
